# The value of vital sign trends in predicting and monitoring clinical deterioration: A systematic review

**DOI:** 10.1371/journal.pone.0210875

**Published:** 2019-01-15

**Authors:** Idar Johan Brekke, Lars Håland Puntervoll, Peter Bank Pedersen, John Kellett, Mikkel Brabrand

**Affiliations:** 1 Department of Clinical Research, University of Southern Denmark, Odense, Denmark; 2 Department of Emergency Medicine, Odense University Hospital, Odense, Denmark; 3 Department of Emergency Medicine, Hospital of South West Jutland, Esbjerg, Denmark; 4 Department of Regional Health Research, University of Southern Denmark, Odense, Denmark; University of Notre Dame Australia, AUSTRALIA

## Abstract

**Background:**

Vital signs, i.e. respiratory rate, oxygen saturation, pulse, blood pressure and temperature, are regarded as an essential part of monitoring hospitalized patients. Changes in vital signs prior to clinical deterioration are well documented and early detection of preventable outcomes is key to timely intervention. Despite their role in clinical practice, how to best monitor and interpret them is still unclear.

**Objective:**

To evaluate the ability of vital sign trends to predict clinical deterioration in patients hospitalized with acute illness.

**Data Sources:**

PubMed, Embase, Cochrane Library and CINAHL were searched in December 2017.

**Study Selection:**

Studies examining intermittently monitored vital sign trends in acutely ill adult patients on hospital wards and in emergency departments. Outcomes representing clinical deterioration were of interest.

**Data Extraction:**

Performed separately by two authors using a preformed extraction sheet.

**Results:**

Of 7,366 references screened, only two were eligible for inclusion. Both were retrospective cohort studies without controls. One examined the accuracy of different vital sign trend models using discrete-time survival analysis in 269,999 admissions. One included 44,531 medical admissions examining trend in Vitalpac Early Warning Score weighted vital signs. They stated that vital sign trends increased detection of clinical deterioration. Critical appraisal was performed using evaluation tools. The studies had moderate risk of bias, and a low certainty of evidence. Additionally, four studies examining trends in early warning scores, otherwise eligible for inclusion, were evaluated.

**Conclusions:**

This review illustrates a lack of research in intermittently monitored vital sign trends. The included studies, although heterogeneous and imprecise, indicates an added value of trend analysis. This highlights the need for well-controlled trials to thoroughly assess the research question.

## Introduction

Vital signs, including respiratory rate, oxygen saturation, blood pressure, pulse and temperature, are the simplest, cheapest and probably most important information gathered on hospitalized patients [1]. However, despite being introduced into clinical practice more than a century ago, surprisingly few attempts have been made to quantify their clinical performance [2]. In the last few decades, vital signs have become an area of active research [1] and numerous studies have reported that changes in vital signs occur several hours prior to a serious adverse event [3–7].

Today, vital signs play an important role in emergency departments (ED) and on the wards, to determine patients at risk of deterioration [6–11]. Even though it is accurately predicted by vital sign changes, clinical deterioration often goes unnoticed, or is not detected until it is too late to treat [12–15]. This is mainly caused by inadequate recording of vital signs or as a result of an inappropriate response to abnormal values [1, 14–16]. Among nurses and doctors there is insufficient knowledge and appreciation of vital sign changes and their implications for patient care [17–20]. The importance of monitoring vital signs in clinical practice is indisputable, but how to best monitor and interpret them and how frequently they should be measured is still unclear [21, 22].

This review searched the literature for studies that explicitly tried to determine and quantify the increase or decrease in risk associated with changes of intermittently measured vital signs. We, therefore, confined our search only to those papers that measured vital signs intermittently, and not to those that used continuous monitoring and novel wearable technology

## Methods

### Objective

The aim of this systematic review was to evaluate the ability of intermittent vital sign trends to predict clinical deterioration in acutely ill patients in hospital.

### Protocol and registration

The protocol for this review was registered in PROSPERO: CRD42017080303. Both the protocol and the article are developed in accordance with the Preferred Reporting Items for Systematic Reviews and Meta-Analysis guidelines (S1 Appendix) [23, 24].

### Eligibility criteria

Inclusion criteria: all studies based on intermittent vital sign trends in acutely ill adult patients on hospital wards and in EDs, including all observational studies and controlled trials assessing prognosis. Trends were defined as the changes between two or more consecutive measurements of vital sign values, with a minimum of 3 hours and a maximum of 24 hours between measurements. Articles in English, Danish, Norwegian or Swedish were included.

Exclusion criteria: case series and case reports, studies on patients with specific conditions or with less than 100 participants, or patients directly admitted to ICU. All studies reporting trends in continuous monitoring were excluded.

Outcomes: in-hospital mortality or mortality up to 30 days after hospital discharge, transfer to ICU, cardiac arrest, calls to a rapid response system, or any other outcome reported that was associated with clinical deterioration.

### Information sources

We searched PubMed, Embase, Cochrane Library and CINAHL on October 26th 2017. The databases were searched without time restrictions or filters for language and study design. The search was updated on December 28th 2017, adding the term “trajectory” to the original search (S2 Appendix). PROSPERO was searched for relevant ongoing or recently completed systematic reviews, last on December 18th. All studies assessed in full-text were screened for relevant citing articles using Scopus and Web of Science (S3 Appendix). Experts in the field were contacted to identify additional relevant studies.

### Search

The search strategy was developed through a series of preliminary searches using a broad range of relevant keywords and thesauri, including; vital sign, deterioration and trend (S2 Appendix). An information specialist from the Medical Research Library at University of Southern Denmark reviewed the search strategy before the final searches were conducted.

### Study selection

Reference handling and duplicate screening was performed using EndNote and Covidence. After removal of duplicates, titles and abstracts were screened independently by two authors (LHP and IJB). Disagreements regarding inclusion were resolved through discussion. In case of continued disagreement, inclusion was decided by a third author.

Eligible studies were read in full length by LHP and IJB and separately assessed against inclusion and exclusion criteria decided by all authors (S4 Appendix). Disagreements were discussed with the other authors, and consensus decided inclusion.

### Data collection process

Data from included studies were extracted separately by LHP and IJB using a preformed data extraction sheet. Collection included: study characteristics, settings, demographics, intervention details and outcomes.

### Risk of bias in individual studies

Critical appraisal was performed in duplicates by the two reviewers. Neither of the authors were blinded. The Quality in Prognosis Studies (QUIPS) tool for prognostic studies [25] was used to evaluate the included studies. The risk of bias was rated within six domains: study participation, study attrition, prognostic factor measurement, outcome measurement, study confounding and statistical analysis and reporting, assessing the risk of bias as either high, moderate or low.

### Risk of bias across studies

The certainty of evidence was evaluated inspired by the Grading of Recommendations Assessment (GRADE) [26]. GRADE is originally designed to evaluate the certainty of evidence in randomized controlled trials. The approach assesses the strength of the body of evidence within five domains: within-study risk of bias (QUIPS), directness, heterogeneity, precision of effect estimates and risk of publication bias. An overall judgement regarding the certainty of the evidence was awarded for each examined outcome, as high, moderate, low or very low. As our study was observational by nature and did not address effect, evidence was not upgraded based on standard criteria. LHP and IJB evaluated the studies independently. Results were compared and discussed with the other authors.

## Results

### Study selection and characteristics

**Fig 1:**
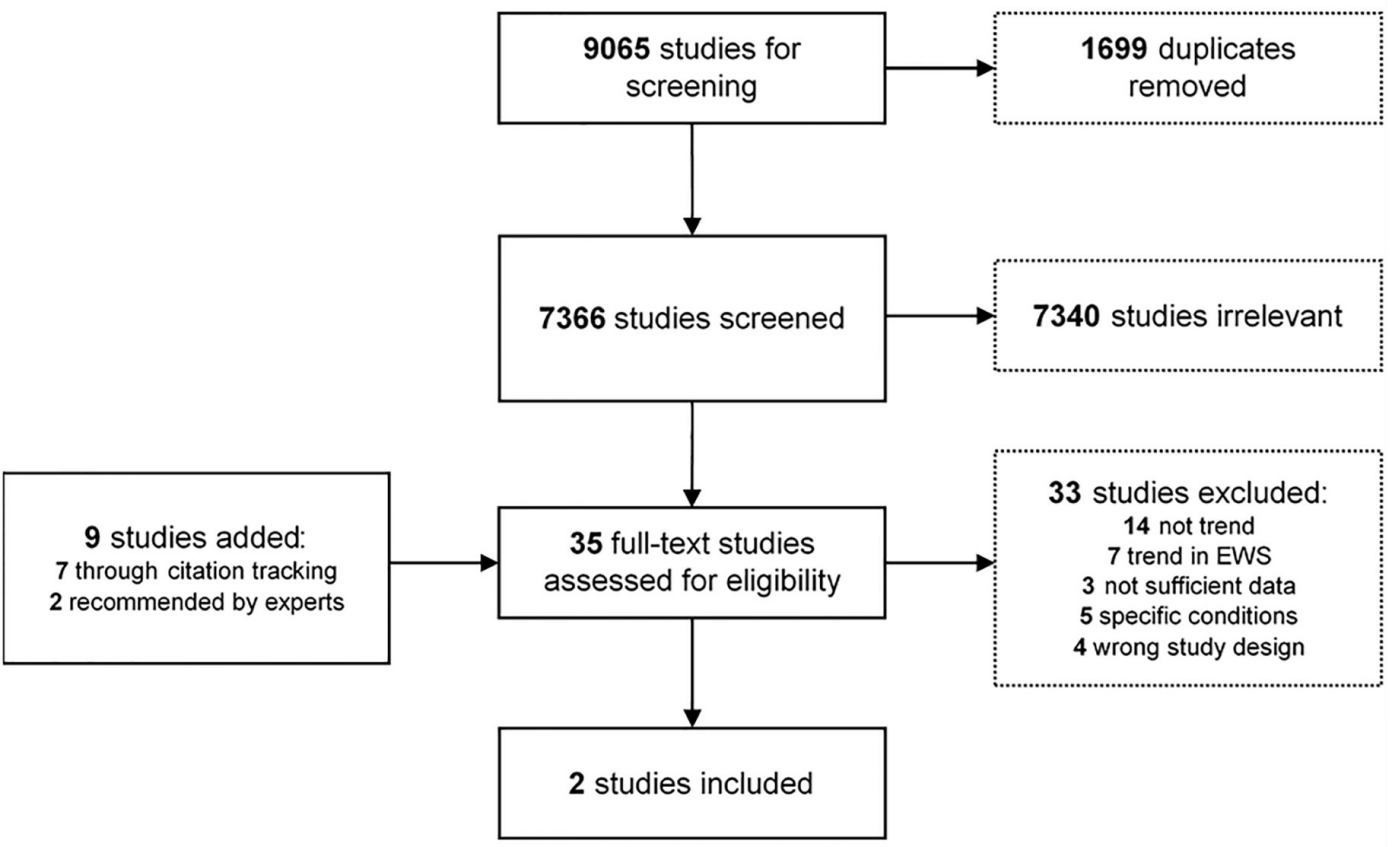
Flowchart of study selection. Abbreviation: EWS–early warning score.

The final search yielded 7,366 studies after removal of duplicates. However, 7,340 were deemed irrelevant (Fig 1). Twenty-six were read in full-text and another nine were added through other sources: seven through citation tracking [27–33] and two additional studies recommended by experts [34, 35]. Of the thirty-five studies assessed, two were eligible for inclusion. Details of the study selection are presented in Fig 1.

We excluded thirty-three studies assessed in full-text. Twenty-five, as they did not fulfil our eligibility criteria; fourteen did not examine trend, seven focused on trends in clinical scoring systems and three incorporated elements of vital sign trends in multi-parameter risk stratification models, but did not present sufficient data to enable analysis. Five studies examined trends in vital signs or EWS in patients with specific conditions and four studies were excluded due to wrong study design. Reasons for exclusion and details are given in S4 Appendix.

We found two cohort studies eligible for inclusion. One including 269,999 medical and surgical admissions in five hospitals in Illinois, by Churpek et al. [36] and one including 44,531 medical admissions to a Canadian regional hospital, by Kellett et al. [37]. Both were retrospective analyses of vital signs collected in electronic medical records that included: respiratory rate, heart rate, systolic and diastolic blood pressure, temperature and oxygen saturation.

Churpek et al. aimed to compare the accuracy of different methods of modelling vital sign trends for detecting clinical deterioration on the wards using discrete-time survival analysis. Six different trend models were tested against the predictive value of current vital signs alone (Table 1). Transfers to intensive care unit (ICU), cardiac arrests and deaths on the ward were analysed as a composite outcome. Vital signs were averaged for each four-hour time block, and the variables at the beginning on each interval used to predict risk of deterioration during that time block.

**Table 1:** Study characteristics.

**Study**	**Design and setting**	**Participants**	**Interventions**	**Outcomes**	**Results**
Churpek et al. [36] 2016 United States Conflicts of interest: Declared	Retrospective cohort study All ward admissions at five hospitals in Illinois between: November 2008—January 2013 Analysis of manually collected vital signs, documented electronically: heart rate respiration rate oxygen saturation temperature systolic blood pressure diastolic blood pressure	All 269,999 ward admissions Age: 60 years (SD 20) Women: 60% White: 52% Length of stay: NS	Vital signs collected on average every 4 h analysed using discrete-time survival analysis. Variables at the beginning on each 4 h interval used to predict risk of event during that time block. Trend variables investigated: change in current value from previous value (delta) mean of the previous six values (mean) standard deviation of the previous six values (SD) slope of the previous six values (slope) minimum value prior to current value (min) maximum value prior to current value (max) exponential smoothing method (smoothed) 60% of the dataset used model design, 40% for validation of accuracy. Univariate analysis of current vital sign and the different trend models, followed by bivariate modelling including both current value and trend value.	Development of critical illness on the wards. Composite outcome, 16,452 (6.1%): 2840 (1.0%) deaths on the ward 424 (0.16%) ward cardiac arrest 13188 (4.9%) ICU transfers Only first outcome examined. In case of multiple ward stays during same admission, each stay analysed separately. Each ICU-stay analysed separately.	Univariate analysis, AUC: Current respiratory rate: 0.70 (95% CI 0.70–0.70) SD respiratory rate: 0.71 (95% CI 0.71–0.71) Current oxygen saturation: 0.59 (95% CI 0.59–0.59) Min oxygen saturation: 0.60 (95% CI 0.60–0.60) Current heart rate: 0.63 (95% CI 0.63–0.64) Current systolic blood pressure: 0.61 (95% CI NS) Current temperature: 0.57 (95% CI NS) Bivariate analysis, AUC (95% CI NS): Max respiratory rate: 0.73 Min respiratory rate: 0.69 Min oxygen saturation: 0.63 Slope/delta oxygen saturation: 0.57 Slope heart rate: 0.66 Delta heart rate: 0.63 Slope systolic blood pressure: 0.64 Delta/smoothed systolic blood pressure: 0.61 SD/slope/delta temperature: 0.58 Smoothed/mean/max/min temperature: 0.57
Kellett et al. [37] 2015 Canada Conflicts of interest: None declared	Retrospective cohort study All medical admissions at Thunder Bay Regional Health Sciences Centre in Ontario between: January 1st 2005—June 30th 2011 Analysis of manually collected vital signs, documented electronically: heart rate respiration rate oxygen saturation temperature systolic blood pressure diastolic blood pressure	44,531 medical admissions of 18,531 patients Age: 67.5 (SD 17.9) Gender: NS Length of stay: 9.5 days (SD 14.5)	Each individual vital sign assigned a weighted ViEWS-score and averaged for every 24 h of admission. Average number of measurements per patient each day: Highest: Heart rate 3.3 Lowest: Temperature 2.4 Change in ViEWS weighted score average between first 5 and last 5 days of admission.	In-hospital mortality: 2067 (4.6%) died within 30 days of admission. Age: 74.5 (SD 14.5) Length of stay: 8.1 days (SD 7.2)	Survived 30 days: Heart rate On admission: 0.24 (SD 0.50) At discharge: 0.15 (SD 0.39) Breathing rate On admission: 0.24 (SD 0.71) At discharge: 0.10 (SD 0.49) Breathing rate + Oxygen saturation: On admission: 0.28 (SD 0.52) At discharge: 0.21 (SD 0.43) Died in hospital (within 30 days): Heart rate On admission: 0.58 (SD 0.74) At discharge: 0.84 (SD 0.88) Breathing rate On admission: 0.92 (SD 1.22) At discharge: 1.46 (SD 1.34) Breathing rate + Oxygen saturation: On admission: 0.80 (SD 0.85) At discharge: 1.30 (SD 0.97)

Abbreviations: NS–not specified, AUC–area under curve, ViEWS–VitalPac early warning score, ICU–intensive care unit

Kellett et al. aimed to assess whether changes in vital signs would enable detection of in-hospital mortality. They assigned a weighted Vitalpac Early Warning Score (ViEWS) to each vital sign and averaged the score for each twenty-four hour period of admission. Change in mean score between the first five and the last five days of admission were then compared for survivors and non-survivors. Further study characteristics are given in Table 1.

### Risk of bias within included studies

None of the studies accounted for loss to follow-up and no clear assessment of confounders were stated. Statistical analyses varied substantially and the overall risk of bias was rated as moderate for both studies, S5 Appendix.

### Risk of bias across studies

As both studies are observational, the certainty of the evidence was regarded as low. With only one article per outcome, inconsistency was not evaluated. We found no serious indirectness in the studies and publication bias was not suspected. Therefore, Churpek et al. received an overall low rating, while Kellett et al. was downgraded to very low, due to serious imprecision. See S5 Appendix for full description.

### Results of individual studies

Churpek et al. performed univariate analysis of the different trend models and the current value, followed by bivariate analysis combining the trend models with the current value. Through univariate analysis, they found respiratory rate to be the best predictor of deterioration when using the current value, AUC 0.70 (95% CI 0.70–0.70). Standard deviation of respiratory rate was found to be more accurate than the current value (AUC 0.71 (95% CI 0.71–0.71)). Bivariate analyses increased accuracy for all vital signs compared to the current value alone, but the optimal method varied for the different vital signs. The model including the current respiratory rate and the maximum rate prior to current was the most the accurate predictor (AUC 0.73). When averaging the change in accuracy for all vital signs, vital sign slope resulted in the greatest increase (AUC improvement 0.013), while the change from previous value resulted in an average decrease of model accuracy (AUC -0.002).

Analysing trajectories in ViEWS weighted vital signs for the first five and the last five days of admission, Kellett et al. found that the score for respiratory rate increased the most in non-survivors (0.92 (SD 1.22)–1.46 (SD 1.34)) and decreased the most in survivors (0.24 (SD 0.71)–0.10 (SD 0.49)). Combining respiratory rate with other vital signs was not more accurately associated with in-hospital mortality. Due to large standard deviations, none of the vital sign trends were statistically significant.

The heterogeneity between the two studies was high. Apart from methodology and outcomes, the cohorts differed in several ways: Churpek et al. looked at both medical and surgical ward patients, with an unspecified number of elective surgical patients. Average age was 60 years and in-hospital mortality was 1.0%. Kellett et al. looked at medical admissions, with an average age of 67.5 years and an in-hospital mortality of 4.6%, Table 1.

The literature search also identified seven studies on trends in EWS. The results of four studies, otherwise eligible for inclusion, were evaluated and summarized in Table 2. The remaining three studies were based on data from the same cohort as Kellett et al [37].

**Table 2:** Studies on trends in early warning scores.

Study	Design and setting	Participants	Interventions	Outcomes	Results^a^^,^^b^
Groarke et al. [38] 2008 Ireland	Prospective single center cohort study of consecutive admissions over a 30-day period.	225 medical admissions between 8:00 and 19:00. Mean age: 64.7 (SD 19.1) 116 male:109 female	EWS calculated upon arrival and transfer from the MAU to the wards. On average after 5 hours. EWS: Vital signs and mental state.	ICU/CCU-admission Cardiac arrest Length of stay In-hospital mortality	Patients with an improvement in score prior to transfer had the lowest risk of reaching any of the combined outcomes (OR 2.56, CI 1.11 to 5.89, p = 0.028).
Kellett et al. [39] 2011 Ireland	Prospective single center cohort study of consecutive medical admissions over a one year period.	1165 medical admissions with two reported SCS. Mean age: 65.7 (SD 18.6)	SCS calculated upon arrival and the following day, in average 25 hours (SD 15.8) apart. SCS: Vital signs, mental state, ECG, specific symptoms and prior conditions.	Length of stay In-hospital mortality	Increases in SCS the day after admission was associated with a tenfold increase ((10% vs. 1.1%, OR 10.1, p<0.001) of in-hospital mortality. Low SCS risk patients were just as likely to have a SCS increase as high risk patients.
Kellett et al. [29] 2013 Canada	Retrospective single center cohort study of surgical admissions over a 6 year period.	15,230 patients with two or three (13,098) complete sets of vital signs collected within first 24 hours of admission. Mean age: 55.8 (SD 18.7)	Changes in the first three abbreviated ViEWS recordings. In average 6–12 hours apart. Abbreviated ViEWS: Vital signs.	Length of stay In-hospital mortality	Patients with an initial score of ≥ 3 had a significantly higher overall in-hospital mortality (p<0.0001). Of these patients, those with a lower second score had a significantly lower in-hospital mortality than those with an unchanged score (p<0.001).
Wang et. al. [40] 2017 USA	Retrospective single center cohort study of consecutive RRT activations within 48h of admission to hospital over a 9 month period.	161 RRT activations during the first 48 hours of admission. 0–12 hours: 20,5% 12–24 hours: 29,8% 24–48 hours: 49,7% Mean age: 64 (SD 20) 104 female:57 male	Functional status, comorbidity, and severity of illness (MEWS and APACHE-2 scoring systems). MEWS: Vital signs, mental state, urine output APACHE-2: Vital signs, mental state, paraclinical measures.	ICU-consult/transfer Palliative care consult Changes in health care directions	MEWS and APACHE-2 scores were higher at the time of RRT activation compared to scores at hospital admission (p<0.0001), but was not associated with increased likelihood of ICU-consultation or acceptance.

Abbreviations: EWS–early warning score, MAU–medical admission unit, ICU–intensive care unit, CCU–coronary care unit, SCS–simple clinical score, ECG–electrocardiography, ViEWS–VitalPac early warning score, RRT–rapid response team, MEWS–modified early warning score. APACHE-2 –acute physiology and chronic health evaluation II. Vital signs: Heart rate, respiratory rate, oxygen saturation, blood pressure (systolic or mean arterial) and temperature.

^a^ For all scoring systems: A higher score equals more deranged vital signs.

^b^ Risk of bias was assessed with QUIPS and GRADE. All studies were evaluated to have a moderate risk of bias and a very low certainty of evidence (S5 Appendix).

## Discussion

This systematic review looked at trends in intermittently monitored vital signs and identified two studies eligible for inclusion. Both examined intermittent vital sign trends as an independent predictor of clinical deterioration. Although largely heterogeneous, with a low certainty of evidence, they suggested trends to be associated with deterioration.

Churpek et al. found respiratory rate to be the most accurate predictor, both for current value and when adding trend models. The most accurate model varied between the vital signs. Although trend statistically increased model accuracy for all vital signs, the improvements were considered minor. Kellett et al. suggested a correlation between increasing ViEWS weighted vital signs and in-hospital mortality. Similarly to Churpek et al., they found respiratory rate to be best associated with outcome, with the largest increase in score for non-survivors and decrease for survivors. However, due to large standard deviations, their findings were not statistically significant.

In essence, both studies suggest that more precise prognostic information can be obtained from changes in vital signs if they undergo manipulation. Kellett et al suggested that the values should be weighted, and Churpek et al found that the difference from the current and previous value was less valuable than the vital sign slope, vital sign variability, and the most deranged values since admission. Their findings also illustrates the lack of consensus in what constitutes trends, and how to best interpret them.

Considering vital signs central role in daily clinical practice, their results, although only suggestive, should be of interest to clinicians caring for patients on wards or in EDs. A lot of effort is going into developing continuous monitoring on the assumption that the trends it will reveal will be clinically valuable and superior to intermittent monitoring [41]. Although considering the technology promising, three recent systematic reviews did not find sufficient evidence in to support the implementation of routinely continuous monitoring of vital signs in general wards [42–44]. Results of this systematic review suggest that combining the widespread use of electronic healthcare systems to record intermittently monitored vital signs with trend analysis could improve the prediction of deterioration prior to a serious adverse event and help direct limited resources towards the patients at risk.

As illustrated by this review, there is an apparent lack of high quality evidence regarding trends in intermittently monitored vital signs. The studies included are retrospective analyses of pre-existing cohorts, without control groups, and with complete heterogeneity. Thus, they have a low (or very low) certainty of evidence. Interestingly, both studies found respiratory rate to be best associated with clinical course, a standpoint receiving a growing support [1, 44, 45]. Currently, there is no reliable and convenient way to evaluate respiratory rate, but recent technological advances will soon enable automated monitoring of respiratory rate [2, 44], and can prove to be a major advance in monitoring. Ultimately, both trends in vital signs in general and respiratory rate in particular, should be subjected to evaluation through well-controlled prospective multicentre cohort studies.

Several studies examining trajectories of intermittently monitored vital signs were not eligible for inclusion (S4 Appendix). These consisted of; risk stratification models with elements of vital sign trends, trends in EWS and in patients with specific conditions, including; cardiac arrest [46], advanced stage of cancer [34], acute respiratory condition [47], repeated emergency team activations [48] and normotensive ED patients [31]. Although not subject for inclusion, they are mentioned to give an account of the total number of studies on vital sign trends identified by the review.

Likewise, studies on trends in EWS, otherwise meeting the inclusion criteria, are listed in Table 2, in order to make the review more informative. They illustrate a potential correlation between trends and clinical deterioration. As observational studies with small sample sizes and low number of events, their findings should be interpreted with caution. They were all evaluated to have a moderate risk of bias and a very low certainty of evidence.

However, there are multiple limitations to such risk stratification models. In a recent article, Baker & Gerdin [49] discussed the clinical usefulness of the large number of prediction models developed for use in critical care. They emphasised the current focus on trying to optimise the precision of these models, rather than testing the performance of the models to real-world interventions and their impact on outcomes. Similarly, Pedersen et al. [10] highlighted the need to evaluate the endpoints currently used to validate these predictive models (e.g. ICU-transfer, cardiac arrest and in-hospital mortality). They argued for the importance of developing systems that specifically can identify patients who are salvageable, if provided with optimal treatment and care.

Disappointingly, only two studies were found eligible for inclusion in this review of intermittently monitored vital sign trends. Still, the fact that there is little or no high quality evidence supporting trends in vital signs and the myriads of scoring systems developed to the means of predicting clinical deterioration, should be an essential contribution to evidence based practice.

### Strengths

The search strategy was developed for a high sensitivity, with the aim of identifying all studies examining trend, without filtering for time or language. An information specialist reviewed the search strategy before the final searches were conducted. Only studies examining continuous monitoring were excluded on time criteria, in the abstract screening. Hence, changing the minimum time to 1 hour would not yield any further eligible studies. Reference tracking and outreach to relevant experts did not identify any other eligible studies that were not identified by the original search.

### Limitations

This review only descriptively analysed the eligible studies identified and did not quantify data or perform a meta-analysis. Due to the wide applicability of the search terms “vital signs” and “trend”, only a small number of the articles were deemed relevant and assessed in full text. To reflect the clinical ward setting, the protocol for the review narrowed the inclusion criteria to studies analysing trends with a minimum of 3 hours and a maximum of 24 hours between measurements [21, 22]. The evidence supporting measurement frequency is limited at best, and as a result, no studies were excluded on this criterion alone during abstract screening. Apart from reference tracking and expert outreach, attempts to pursue grey literature were not made.

## Conclusions

The two eligible studies identified suggest that trend analysis of intermittent vital signs would increase the accuracy for detection of clinical deterioration on general wards and in EDs. However, the external validity of these findings is challenging to test–and there is a need to shift the focus towards clinical feasibility. Furthermore, the results of this review show there is no consensus on how to best analyse trends. Given that trend-models are externally validated through well-controlled prospective multicentre cohort studies, authors of this review, consider them promising and welcome as a valuable addition to clinical decision support.

## Supporting information

S1 Appendix PRISMA 2009 checklist. (DOCX)

S2 Appendix Search strategy. (DOCX)

S3 Appendix Citation tracking. (DOCX)

S4 Appendix Full-text screening. (DOCX)

S5 Appendix Risk of bias assessment. (DOCX)
