# Supplementary material for: The value of vital sign trends in predicting and monitoring clinical deterioration: A systematic review
Source: PLoS One. 2019 Jan 15;14(1):e0210875. doi: 10.1371/journal.pone.0210875 (PMC6333367; doi:10.1371/journal.pone.0210875)
Supplement: S2 Appendix — (DOCX) [file pone.0210875.s002.docx]

S2 Appendix: Search strategy

**Facet 1**

|  | **PubMed/Cochrane** | **Embase** | **CINAHL** |  |
| --- | --- | --- | --- | --- |
| Vital sign |  | Thesaurus |  |  |
| Vital signs | MeSH term |  | Heading |  |
| Vital value |  |  |  |  |
| Vital values |  |  |  |  |
| EWS |  |  |  |  |
| Early warning score |  |  |  |  |
| Early warning scores |  |  |  |  |
| Early warning system |  |  |  |  |
| Early warning systems |  |  |  |  |
| Track and trigger |  |  |  |  |
| Track-and-trigger |  |  |  |  |
| Scoring system |  | Thesaurus |  |  |
| Scoring systems |  |  |  |  |
| Physiologic monitoring | MeSH term | Thesaurus | Heading |  |
| Physiological parameters |  | Thesaurus |  |  |
| Early diagnosis | MeSH term | Thesaurus | Heading |  |
| Triage | MeSH term |  | Heading |  |
| Triages |  |  |  |  |
| Risk assessment | MeSH term | Thesaurus | Heading |  |
| Risk assessments |  |  |  |  |
| Risk adjustment | MeSH term | Thesaurus |  |  |
| Risk Adjustments |  |  |  |  |
| Severity of Illness Index | MeSH term | Thesaurus |  |  |
| Severity of illness indices |  |  | Heading |  |
| Severity of disease index |  |  |  |  |
| Severity of disease indicies |  |  |  |  |
| Decision support system |  | Thesaurus |  |  |
| Decision support systems |  |  |  |  |
| Clinical Decision Support Systems | MeSH term | Thesaurus | Heading |  |
| Decision support technique |  |  |  |  |
| Decision Support Techniques | MeSH term |  | Heading |  |
| AND |  |  |  |  |
| Respiratory rate | MeSH term |  | Heading |  |
| Respiratory frequency |  |  |  |  |
| Breathing rate |  | Thesaurus |  |  |
| Breathing frequency |  |  |  |  |
| Respiration rate |  |  |  |  |
| Respiration frequency |  |  |  |  |
| Heart rate | MeSH term | Thesaurus | Heading |  |
| Heart frequency |  |  |  |  |
| Cardiac rate |  |  |  |  |
| Cardiac frequency |  |  |  |  |
| Ventricle rate |  |  |  |  |
| Body temperature | MeSH term | Thesaurus | Heading |  |
| Body temperatures |  |  |  |  |
| Blood pressure | MeSH term | Thesaurus | Heading |  |
| Blood pressures |  |  |  |  |
| Systolic pressure |  |  |  |  |
| Diastolic pressure |  |  |  |  |

**Facet 2**

|  | **PubMed/Cochrane** | **Embase** | **CINAHL** |  |
| --- | --- | --- | --- | --- |
| Deterioration |  | Thesaurus |  |  |
| Deteriorating |  |  |  |  |
| Prognosis | MeSH Term | Thesaurus | Heading |  |
| Prognostic |  |  |  |  |
| Prognostic assessment |  | Thesaurus |  |  |
| Disease progression | MeSH Term |  | Heading |  |
| Disease course |  | Thesaurus |  |  |
| Clinical course |  |  |  |  |
| Illness trajectory |  | Thesaurus |  | Moved to facet 3 in updated search 28.12.2017 |
| Trajectory |  |  |  |  |
| Trajectories |  |  |  |  |
| Variation |  |  |  |  |
| Variability |  |  |  |  |
| Hospital admission |  | Thesaurus |  |  |
| Length of stay | MeSH Term | Thesaurus | Heading |  |
| Survival rate | MeSH Term | Thesaurus |  |  |
| Survival rates |  |  |  |  |
| Outcome |  |  |  |  |
| Outcomes |  |  |  |  |
| Heart arrest | MeSH term | Thesaurus | Heading |  |
| Asystole |  |  |  |  |
| Asystolia |  |  |  |  |
| Asystoly |  |  |  |  |
| Cardiac arrest |  |  |  |  |
| Circulation arrest |  |  |  |  |
| Circulatory arrest |  |  |  |  |
| Heart asystole |  |  |  |  |
| Heart standstill |  |  |  |  |
| Cardiopulmonary arrest |  |  |  |  |
| Death | MeSH term | Thesaurus | Heading |  |
| Mortality | MeSH term | Thesaurus | Heading |  |
| Hospital Rapid Response Team | MeSH term |  |  |  |
| Rapid response team |  | Thesaurus | Heading |  |
| Rapid response teams |  |  |  |  |
| Rapid response system |  |  |  |  |
| Rapid response systems |  |  |  |  |
| Medical emergency team |  |  |  |  |
| Medical emergency teams |  |  |  |  |
| Outreach service |  |  |  |  |

**Facet 3**

|  | **PubMed/Cochrane** | **Embase** | **CINAHL** |  |
| --- | --- | --- | --- | --- |
| Trend |  |  |  |  |
| Trends |  |  |  |  |
| Trend study |  | Thesaurus |  |  |
| Trend studies |  |  | Heading |  |
| Interrupted Time Series Analysis | MeSH term |  | Heading |  |
| Time series analysis |  | Thesaurus |  |  |
| Time series |  |  | Heading |  |
| Trajectory |  |  |  | Added in updated search 28.12.2017 |
| Trajectories |  |  |  |  |
| Illness trajectory |  | Thesaurus |  |  |

**PubMed**

|  | **Search** | **26.10.2017** | **28.12.2017** |  |
| --- | --- | --- | --- | --- |
| #1 | Vital Signs[MeSH Terms] | 382016 | 384868 |  |
| #2 | "Vital Sign" OR "Vital Signs" OR "Vital Value" OR "Vital Values" OR EWS OR "Early Warning Score" OR "Early Warning Scores" OR "Early Warning System" OR "Early Warning Systems" OR "Track and Trigger" | 14735 | 15247 |  |
| #3 | Physiological monitoring[MeSH Terms] | 152853 | 155129 |  |
| #4 | Early diagnosis[MeSH Terms] | 37791 | 38950 |  |
| #5 | Triage[MeSH Terms] | 9728 | 9881 |  |
| #6 | Risk adjustment[MeSH Terms] | 2773 | 2838 |  |
| #7 | Risk assessment[MeSH Terms] | 220715 | 225134 |  |
| #8 | Severity of illness index[MeSH Terms] | 213799 | 217695 |  |
| #9 | Clinical decision support systems[MeSH Terms] | 6360 | 6557 |  |
| #10 | Decision Support Techniques[MeSH Terms] | 69134 | 69974 |  |
| #11 | "Scoring system" OR "Scoring systems" OR "Physiological monitoring" OR "Physiological parameters" OR "Early diagnosis" OR "Triage" OR "Triages" OR "Risk adjustment" OR "Risk adjustments" OR "Risk assessment" OR "Risk assessments" OR "Severity of illness index" OR "Severity of illness indices" OR "Severity of disease index" OR "Severity of disease indices" OR " Decision support system" OR "Decision Support Systems" OR "Decision support technique" OR "Decision support techniques" | 593666 | 604392 |  |
| #12 | #3 OR #4 OR #5 OR #6 OR #7 OR #8 OR #9 OR #10 OR #11 | 803639 | 817511 |  |
| #13 | Respiratory Rate[MeSH Terms] | 2021 | 2085 |  |
| #14 | Heart Rate[MeSH Terms] | 157330 | 158586 |  |
| #15 | Body Temperature[MeSH Terms] | 81211 | 81649 |  |
| #16 | Blood Pressure[MeSH Terms] | 287261 | 289374 |  |
| #17 | "Respiratory rate" OR "Respiratory frequency" OR "Breathing rate" OR "Breathing frequency" OR "Respiration rate" OR "Respiration frequency" OR "Heart rate" OR "Heart frequency" OR "Cardiac rate" OR "Cardiac frequency" OR "Ventricle rate" OR "Body temperature" OR "Body temperatures" OR "Blood pressure" OR "Blood pressures" OR "Systolic pressure" OR "Diastolic pressure" | 625460 | 629784 |  |
| #18 | #13 OR #14 OR #15 OR #16 OR #17 | 652242 | 656911 |  |
| #19 | #12 AND #18 | 56729 | 57648 |  |
| **#20** | **#1 OR #2 OR #19** | **419870** | **423571** | **Facet 1** |
| #21 | Prognosis[MeSH Terms] | 1363430 | 1387574 |  |
| #22 | Disease Progression [MeSH Terms] | 147305 | 150593 |  |
| #23 | Length of stay [MeSH Terms] | 73350 | 74672 |  |
| #24 | Survival rate [MeSH Terms] | 149559 | 151776 |  |
| #25 | Heart Arrest[MeSH Terms] | 41124 | 41863 |  |
| #26 | Death[MeSH Terms] | 134901 | 136144 |  |
| #27 | Mortality[MeSH Terms] | 332071 | 336828 |  |
| #28 | Hospital Rapid Response Team[MeSH Terms] | 560 | 582 |  |
| #29 | "Deterioration" OR "Deteriorating" OR "Prognosis" OR "Prognostic" OR "Disease progression" OR "Disease course" OR "Clinical course" OR "Trajectory" OR "Trajectories" OR "Variation" OR "Variability" OR "Hospital admission" OR "Length of stay" OR "Survival rate" OR "Survival rates" OR "Outcome" OR "Outcomes" OR "Heart arrest" OR "Asystole" OR "Asystolia" OR "Asystoly" OR "Cardiac arrest" OR "Circulation arrest" OR "Circulatory arrest" OR "Heart asystole" OR "Heart standstill" OR "Cardiopulmonary arrest" OR "Death" OR "Mortality" OR "Hospital Rapid Response Team" OR "Rapid response team" OR "Rapid response teams" OR "Rapid response system" OR "Rapid response systems" OR "Medical emergency team" OR "Medical emergency teams" OR "Outreach service" | 4349372 | 4370087 | Moved to facet 3 in updated search 28.12.2017 |
| **#30** | **#21 OR #22 OR #23 OR #24 OR #25 OR #26 OR #27 OR #28 OR #29** | **4470396** | **4492787** | **Facet 2** |
| #31 | Interrupted Time Series Analysis[MeSH Terms] | 314 | 365 |  |
| #32 | "Trend" OR "Trends" OR "Time Series" OR "Trajectory" OR "Trajectories" | 677191 | 738221 | Added in updated search 28.12.2017 |
| **#33** | **#31 OR #32** | **677191** | **738221** | **Facet 3** |
| **#34** | **#20 AND #30 AND #33** | **4654** | **4939** |  |

**Embase**

|  | **Search** | **26.10.2017** | **28.12.2017** |  |
| --- | --- | --- | --- | --- |
| 1 | Exp Vital sign/ | 16702 | 17126 |  |
| 2 | Vital sign or Vital signs or Vital value or Vital values or EWS or Early Warning Score or Early Warning Scores or Early Warning System or Early Warning Systems or "Track and trigger" | 30650 | 31334 |  |
| 3 | Exp Scoring system/ | 222579 | 225885 |  |
| 4 | Exp Physiologic monitoring/ | 4346 | 4443 |  |
| 5 | Exp Early diagnosis/ | 91420 | 92656 |  |
| 6 | Exp Risk assessment/ | 435838 | 446924 |  |
| 7 | Exp Severity of illness index/ | 10971 | 11275 |  |
| 8 | Exp Decision support system/ | 19502 | 19914 |  |
| 9 | Scoring system or Scoring systems or Physiological monitoring or Physiological parameters or Early diagnosis or Triage or Triages or Risk assessment or Risk assessments or Risk adjustment or Risk adjustments or Severity of illness or Severity of illnesses or Severity of disease or Severity of diseases or Decision support system or Decision support systems or Decision support technique or Decision support techniques | 894560 | 912929 |  |
| 10 | 3 or 4 or 5 or 6 or 7 or 8 or 9 | 898464 | 916925 |  |
| 11 | Exp Breathing rate/ | 29663 | 30349 |  |
| 12 | Exp Heart rate/ | 228266 | 231159 |  |
| 13 | Exp Body temperature/ | 55262 | 55854 |  |
| 14 | Exp Blood pressure/ | 508167 | 515050 |  |
| 15 | Breathing rate or Breathing frequency or Respiration frequency or Respiration rate or Respiratory frequency or Respiratory rate or Heart rate or Cardiac frequency or Cardiac rate or Heart frequency or Ventricle rate or Body temperature or Blood pressure or Blood pressures or Systolic pressure or Diastolic pressure | 796657 | 806809 |  |
| 16 | 11 or 12 or 13 or 14 or 15 | 871246 | 882644 |  |
| 17 | 10 and 16 | 41000 | 41966 |  |
| **18** | **1 or 2 or 17** | **70474** | **72086** | **Facet 1** |
| 19 | Exp Deterioration/ | 34624 | 35412 |  |
| 20 | Exp Prognosis/ | 606008 | 616475 |  |
| 21 | Exp Prognostic assessment/ | 7914 | 8415 |  |
| 22 | Exp Disease course/ | 2797355 | 2852422 |  |
|  | Exp Illness trajectory/ |  |  | Moved to facet 3 in updated search 28.12.2017 |
| 23 | Exp Hospital admission/ | 158169 | 161531 |  |
| 24 | Exp Length of stay/ | 139947 | 143831 |  |
| 25 | Exp Survival rate/ | 207090 | 210458 |  |
| 26 | Exp Heart arrest/ | 74882 | 77063 |  |
| 27 | Exp Death/ | 631603 | 642187 |  |
| 28 | Exp Mortality/ | 904422 | 919579 |  |
| 29 | Exp Rapid response team/ | 1641 | 1724 |  |
| 30 | Deterioration or Deteriorating or Prognosis or Prognostic or Disease progression or Disease course or Clinical course or Trajectory or Trajectories or Variation or Variability or Hospital admission or Length of stay or Survival rate or Survival rates or Outcome or Outcomes or Heart arrest or Asystole or Asystolia or Asystoly or Cardiac arrest or Circulation arrest or Circulatory arrest or Heart asystole or Heart standstill or Cardiopulmonary arrest or Death or Mortality or Rapid response team or Rapid response teams or Rapid response system or Rapid response systems or Medical emergency team or Medical emergency teams or Outreach service | 6099277 | 6166846 |  |
| **31** | **19 or 20 or 21 or 22 or 23 or 24 or 25 or 26 or 27 or 28 or 29 or 30** | **7181262** | **7268682** | **Facet 2** |
| 32 | Exp Trend study/ | 21937 | 22832 |  |
| 33 | Exp Time series analysis/ | 20348 | 20753 |  |
| 34 | Exp Illness trajectory/ | 1313 | 1350 | Added in updated search 28.12.2017 |
| 35 | Trend or Trends or Time series or Trajectory or Trajectories | 493449 | 564034 |  |
| 36 | **32 or 33 or 34 or 35** | **493449** | **564034** | **Facet 3** |
| **37** | **18 and 31 and 36** | **1535** | **1666** |  |

**CINAHL**

|  | **Search** | **26.10.2017** | **12.28.2017** |  |
| --- | --- | --- | --- | --- |
| #1 | (MH "Vital Signs+") | 10035 | 10136 |  |
| #2 | "Vital Sign" OR "Vital Signs" OR "Vital Value" OR "Vital Values" OR EWS OR *EWS OR "Early Warning Score" OR "Early Warning Scores" OR "Early Warning System" OR "Early Warning Systems" OR "Track and Trigger" OR "Track-and-trigger" | 5002 | 5073 |  |
| #3 | (MH "Monitoring, Physiologic+") | 51430 | 51859 |  |
| #4 | (MH "Early Diagnosis+") | 5111 | 5276 |  |
| #5 | (MH "Triage+") | 6159 | 6205 |  |
| #6 | (MH "Risk Assessment+") | 43604 | 44127 |  |
| #7 | (MH "Severity of Illness Indices+") | 24754 | 27524 |  |
| #8 | (MH "Decision Support Systems, Clinical+") | 1925 | 1971 |  |
| #9 | (MH "Decision Support Techniques+") | 3747 | 3861 |  |
| #10 | "Scoring system" OR "Scoring systems" OR "Physiological monitoring" OR "Physiological parameters" OR "Early diagnosis" OR "Triage" OR "Triages" OR "Risk adjustment" OR "Risk adjustments" OR "Risk assessment" OR "Risk assessments" OR "Severity of illness index" OR "Severity of illness indices" OR "Severity of disease index" OR " Severity of disease indices" OR " Decision support system" OR "Decision Support Systems" OR "Decision support technique" OR "Decision support techniques" | 91136 | 92265 |  |
| #11 | #3 OR #4 OR #5 OR #6 OR #7 OR #8 OR #9 OR #10 | 144484 | 146133 |  |
| #12 | (MH "Respiratory Rate+") | 1147 | 1156 |  |
| #13 | (MH "Heart Rate+") | 16561 | 16678 |  |
| #14 | (MH "Body Temperature+") | 5662 | 5718 |  |
| #15 | (MH "Blood Pressure+") | 21884 | 22079 |  |
| #16 | "Breathing rate" or "Breathing frequency" or "Respiration frequency" or "Respiration rate" or "Respiratory frequency" or "Respiratory rate" or "Heart rate" or "Cardiac frequency" or "Cardiac rate" or "Heart frequency" or "Ventricle rate" or "Body temperature" or "Blood pressure" or "Blood pressures" or "Systolic pressure" or "Diastolic pressure" | 65231 | 65929 |  |
| #17 | #12 OR #13 OR #14 OR #15 OR #16 | 67552 | 68272 |  |
| #18 | #13 AND #20 | 11903 | 12013 |  |
| #19 | **#1 OR #2 OR #18** | **23459** | **23687** | **Facet 1** |
| #20 | (MH "Prognosis+") | 186894 | 189,37 |  |
| #21 | (MH "Length of Stay+") | 20700 | 20963 |  |
| #22 | (MH "Disease Progression+") | 20195 | 20479 |  |
| #23 | (MH "Heart Arrest+") | 9792 | 9883 |  |
| #24 | (MH "Death+") | 29662 | 29909 |  |
| #25 | (MH "Mortality+") | 37676 | 38114 |  |
| #26 | (MH "Rapid Response Team+") | 17 | 28 |  |
| #27 | "Deterioration" OR "Deteriorating" OR "Prognosis" OR "Prognostic" OR "Disease Progression" OR "disease course" OR "Clinical course" OR "Trajectory" OR "Trajectories" OR "Variation" OR "Variability" OR "Hospital admission" OR "Length of stay" OR "Survival rate" or "Survival rates" OR "Outcome" OR "outcomes" OR "Heart arrest" OR "asystole" OR "asystolia" OR "asystoly" OR "Cardiac arrest" OR "circulation arrest" OR "circulatory arrest" OR "heart asystole" OR "heart standstill" OR "Cardiopulmonary arrest" OR "Death" OR "Mortality" OR "Hospital Rapid Response Team" OR "Rapid response team" OR "Rapid response teams" OR "Rapid response system" OR "Rapid response systems" OR "medical emergency team" OR "medical emergency teams" OR "Outreach service" | 651731 | 656653 | Moved to facet 3 in updated search 28.12.2017 |
| #28 | **#20 OR #21 OR #22 OR #23 OR #24 OR #25 OR #26 OR #27** | **660690** | **665718** | **Facet 2** |
| #29 | (MH "Interrupted Time Series Analysis+") | 43 | 47 |  |
| #30 | (MH "Trend Studies+") | 898 | 902 |  |
| #31 | (MH"Time Series+") |  | 135 | Added in updated search 28.12.2017 |
| #32 | "Trend" OR "Trends" OR "Time Series" OR "Trajectory" OR "Trajectories" | 170990 | 179406 |  |
| #33 | **#29 OR #30 OR #31 OR #32** | **170990** | **179406** | **Facet 3** |
| #34 | **#19 AND #28 AND #33** | **554** | **575** |  |

**Cochrane Library**

|  | **Search** | **26.10.2017** | **28.12.2017** |  |
| --- | --- | --- | --- | --- |
| #1 | [mh "Vital Signs"] | 34253 | 34717 |  |
| #2 | "Vital Sign" OR "Vital Signs" OR "Vital Value" OR "Vital Values" OR EWS OR "Early Warning Score" OR "Early Warning Scores" OR "Early Warning System" OR "Early Warning Systems" OR "Track and Trigger" | 5400 | 5562 |  |
| #3 | [mh "Monitoring, Physiologic"] | 11316 | 11597 |  |
| #4 | [mh "Early Diagnosis"] | 1613 | 1670 |  |
| #5 | [mh "Triage"] | 332 | 344 |  |
| #6 | [mh "Risk Adjustment"] | 36 | 38 |  |
| #7 | [mh "Risk Assessment"] | 9520 | 9691 |  |
| #8 | [mh "Severity of illness index"] | 18364 | 18681 |  |
| #9 | [mh "Decision Support systems, Clinical"] | 378 | 388 |  |
| #10 | [mh "Decision Support Techniques"] | 3655 | 3692 |  |
| #11 | "Scoring system" OR "Scoring systems" OR "Physiological monitoring" OR "Physiological parameters" OR "Early diagnosis" OR "Triage" OR "Triages" OR "Risk adjustment" OR "Risk adjustments" OR "Risk assessment" OR "Risk assessments" OR "Severity of illness index" OR "Severity of illness indices" OR "Severity of disease index" OR " Severity of disease indices" OR " Decision support system" OR "Decision Support Systems" OR "Decision support technique" OR "Decision support techniques" | 51460 | 52729 |  |
| #12 | #3 OR #4 OR #5 OR #6 OR #7 OR #8 OR #9 OR #10 OR 11 | 260792 | 258497 |  |
| #13 | [mh "Respiratory rate"] | 201 | 206 |  |
| #14 | [mh "Heart rate"] | 17964 | 18194 |  |
| #15 | [mh "Body temperature"] | 3802 | 3834 |  |
| #16 | [mh "Blood pressure"] |  | 26367 |  |
| #17 | "Respiratory rate" OR "Respiratory frequency" OR "Breathing rate" OR "Breathing frequency" OR "Respiration rate" OR "Respiration frequency" OR "Heart rate" OR "Heart frequency" OR "Cardiac rate" OR "Cardiac frequency" OR "Ventricle rate" OR "Body temperature" OR "Body temperatures" OR "Blood pressure" OR "Blood pressures" OR "Systolic pressure" OR "Diastolic pressure" | 26040 | 88009 |  |
| #18 | #13 OR #14 OR #15 OR #16 OR #17 | 87644 | 89221 |  |
| #19 | #12 AND #18 | 25971 | 25634 |  |
| **#20** | **#1 OR #2 OR #19** | **55002** | **55313** | **Facet 1** |
| #21 | [mh "Prognosis"] | 141718 | 144686 |  |
| #22 | [mh "Disease progression"] | 6711 | 6849 |  |
| #23 | [mh "Length of stay"] | 8011 | 8154 |  |
| #24 | [mh "Survival rate"] | 10017 | 10149 |  |
| #25 | [mh "Heart arrest"] | 1529 | 1570 |  |
| #26 | [mh "Death"] | 1945 | 1975 |  |
| #27 | [mh "Mortality"] | 13443 | 13649 |  |
| #28 | [mh "Hospital rapid response team"] | 14 | 14 |  |
| #29 | Deterioration OR Deteriorating OR Prognosis OR Prognostic OR "Disease progression" OR "Disease course" OR "Clinical course" OR Trajectory OR Trajectories OR Variation OR Variability OR "Hospital admission" OR "Length of stay" OR "Survival rate" OR "Survival rates" OR Outcome OR Outcomes OR "Heart arrest" OR Asystole OR Asystolia OR Asystoly OR "Cardiac arrest" OR "Circulation arrest" OR "Circulatory arrest" OR "Heart asystole" OR "Heart standstill" OR "Cardiopulmonary arrest" OR Death OR Mortality OR "Hospital rapid response team" OR "Rapid response team" OR "Rapid response teams" OR "Rapid response system" OR "Rapid response systems" OR "Medical emergency team" OR "Medical emergency teams" OR "Outreach service" | 411900 | 421343 | Moved to facet 3 in updated search 28.12.2017 |
| **#30** | **#21 OR #22 OR #23 OR #24 OR #25 OR #26 OR #27 OR #28 OR #29** | **415466** | **424959** | **Facet 2** |
| #31 | [mh "Interrupted time series analysis"] | 19 | 20 |  |
| #32 | Trend OR Trends OR "Time Series" OR Trajectory OR Trajectories | 34389 | 47537 | Added in updated search 28.12.2017 |
| **#33** | **#31 OR #32** | **34389** | **57546** | **Facet 3** |
| **#34** | **#20 AND #30 AND #33** | **1927** | **1963** |  |
|  | Cochrane Database of Systematic Reviews : Issue 12 of 12, December 2017 | 946 | 975 | The Cochrane Database of Systematic Reviews (CDSR), 2017 Issue 10 |
|  | Cochrane Central Register of Controlled Trials : Issue 12 of 12, December 2017 | 899 | 910 | The Cochrane Central Register of Controlled Trials (CENTRAL), 2017 Issue 9 |
|  | **Total:** | **1845** | **1885** |  |
