# Supplementary material for: The value of vital sign trends in predicting and monitoring clinical deterioration: A systematic review
Source: PLoS One. 2019 Jan 15;14(1):e0210875. doi: 10.1371/journal.pone.0210875 (PMC6333367; doi:10.1371/journal.pone.0210875)
Supplement: S3 Appendix — (DOCX) [file pone.0210875.s003.docx]

# S3 Appendix: Citation tracking

None = no cited or citing articles relevant for full text screening

# Scopus:

1. Almeida 2017 - Detecting dynamical changes in vital signs using switching Kalman filter
   1. Cited: None
   2. Citing: None (09.10.18)
2. Bruera 2014 - Variations in Vital Signs in the Last Days of Life in Patients With Advanced Cancer
   1. Cited: None
   2. Citing: None (09.10.18)
3. Cei 2009 – In-hospital mortality and morbidity of elderly medical patients can be predicted at admission by the Modified Early Warning Score: A prospective study
   1. Cited: Quarterman 2005
   2. Citing: Kim 2015 (10.01.18)
4. J. Chen 2009 – The impact of introducing medical emergency team system on the documentations of vital signs
   1. Cited: None
   2. Citing: None (10.01.18)
5. L. Chen 2017 - Dynamic and personalized risk forecast in step-down units: Implications for monitoring paradigms
   1. Cited: None
   2. Citing: None (09.10.18)
6. Churpek 2016 – The value of vital sign trends for detecting clinical deterioration on the wards
   1. Cited: Escobar 2012, Mao 2011, Wong 2011
   2. Citing: None (10.01.18)
7. Considine 2013 - Emergency department rapid response systems: The case for a standardized approach to deteriorating patients
   1. Cited: None
   2. Citing: None (10.01.18)
8. Derby - 2017 - Clinical Nurse Specialist-Driven Practice Change: Standardizing Vital Sign Monitoring
   1. Cited: None
   2. Citing: None (10.01.18)
9. Devoe 2016 - Correlation of the predictive ability of early warning metrics and mortality for cardiac arrest patients receiving in-hospital Advanced Cardiovascular Life Support
   1. Cited: None
   2. Citing: None (10.01.18)
10. Eggeman 2017
    1. Cited: None
    2. Citing: None (11.01.18)
11. Escobar 2012 – Early detection of impending physiologic deterioration among patients who are not in intensive care: development of predictive models using data from an automated electronic medical record
    1. Cited: None.
    2. Citing: Churpek 2016 (10.01.18)
12. Goldstein 2015 - Classifying Individuals Based on a Densely Captured Sequence of Vital Signs: An Example using Repeated Blood Pressure Measurements during Hemodialysis Treatment
    1. Cited: None
    2. Citing: None (10.01.18)
13. Groarke 2008 – Use of an admission early warning score to predict patient morbidity and mortality and treatment success
    1. Cited: None
    2. Citing: None (10.01.18)
14. Hudson 2015 – Early identification and management of the unstable adult patient in the emergency department
    1. Cited: None
    2. Citing: None (10.01.18)
15. Kellett 2011 - Who will be sicker in the morning? Changes in the Simple Clinical Score the day after admission and the subsequent outcomes of acutely ill unselected medical patients
    1. Cited: None
    2. Citing: Kellett 2013 (Medical) (10.01.2018)
16. Kellett 2014 – How to follow the NEWS
    1. Cited: Murray 2014
    2. Citing: None (10.01.2018)
17. Kellett 2015 – Trends in weighted vital signs and the clinical course of 44,531 acutely ill medical patients while in hospital
    1. Cited: Murray 2014
    2. Citing: None (10.01.2018)
18. Kellett 2013 - Changes and their prognostic implications in the abbreviated Vitalpac™ Early Warning Score (ViEWS) after admission to hospital of 18,853 acutely ill medical patients
    1. Cited: Kellett 2011
    2. Citing: Murray 2014 (10.01.2018)
19. Kellett 2013 – Changes and their prognostic implications in the abbreviated Vitalpac™ Early Warning Score (ViEWS) after admission to hospital of 18,827 surgical patients.
    1. Cited: None
    2. Citing: Subbe 2013 (10.01.2018)
20. Kim 2015 – Modified early warning score changes prior to cardiac arrest in general wards
    1. Cited: None
    2. Citing: None (10.01.2018)
21. Ljunggren 2016 – The association between vital signs and mortality in a retrospective cohort study of an unselected emergency department population
    1. Cited: None
    2. Citing: None (10.01.2018)
22. Mao 2011 – Medical Data Mining for Early Deterioration Warning in General Hospital Wards
    1. Cited: None
    2. Citing: Churpek 2016 (10.01.2018)
23. McGaughey 2007 – Outreach and Early Warning Systems (EWS) for the prevention of Intensive Care admission and death of critically ill adult patients on general hospital wards
    1. Cited: None
    2. Citing: None (09.01.2018)
24. Murray 2014 – Trajectories of the averaged abbreviated Vitalpac early warning score (AbEWS) and clinical course of 44,531 consecutive admissions hospitalized for acute medical illness.
    1. Cited: Kellett 2013 (Medical)
    2. Citing: Kellett 2014, Kellett 2015 (09.01.2018)
25. Nguyen 2016 - Predicting all-cause readmissions using electronic health record data from the entire hospitalization: Model development and comparison
    1. Cited: None
    2. Citing: None (09.01.2018)
26. O'Connell 2016 – A newly designed observation and response chart's effect upon adverse inpatient outcomes and rapid response team activity
    1. Cited: None
    2. Citing: None (09.01.2018)
27. Puskarich 2015 – Abstract: Persistence of tachycardia and tachypnea are associated with mortality in normotensive emergency department patients admitted to the hospital
    1. Cited: None
    2. Citing: Not found (10.01.18)
    3. Not included as reference, but tracking of this abstract yielded full article (Puskarich 2017)
28. Puskarich 2017 – Association between persistent tachycardia and tachypnea and in-hospital mortality among non-hypotensive emergency department patients admitted to the hospital.
    1. Cited: None
    2. Citing: Not found (09.01.2018)
29. Quarterman 2005 - Use of a patient information system to audit the introduction of modified early warning scoring
    1. Cited: None
    2. Citing: Cei 2009 (11.01.18)
30. Schmidt 2015 – Impact of introducing an electronic physiological surveillance system on hospital mortality
    1. Cited: None
    2. Citing: None (09.01.2018)
31. Still 2017 - Predictors of Second Medical Emergency Team Activation Within 24 Hours of Index Event
    1. Cited: Not found (11.01.18)
    2. Citing: Not found (11.01.18)
32. Subbe 2013 – Not getting better means getting worse - Trends in Early Warning Scores suggest that there might only be a short time span to rescue those threatening to fall off a "physiological" cliff?
    1. Cited: Kellett 2013 (Surgical)
    2. Citing: None (09.01.2018)
33. Zimlichman 2012 – Early recognition of acutely deteriorating patients in non-intensive care units: Assessment of an innovative monitoring technology
    1. Cited: None
    2. Citing: None (09.01.2018)
34. Wang 2017 – Early in-hospital clinical deterioration is not predicted by severity of illness, functional status, or comorbidity.
    1. Cited: None
    2. Citing: None (09.01.2018)
35. Wong 2011 – Does adding risk-trends to survival models improve in-hospital mortality predictions? A cohort study
    1. Cited: None
    2. Citing: Churpek 2016 (09.01.2018)

Web of Science:

1. Almeida 2017 - Detecting dynamical changes in vital signs using switching Kalman filter
   1. Citing: Not found (09.01.18)
2. Bruera 2014 - Variations in Vital Signs in the Last Days of Life in Patients With Advanced Cancer
   1. Citing: None (10.01.18)
3. Cei 2009 – In-hospital mortality and morbidity of elderly medical patients can be predicted at admission by the Modified Early Warning Score: A prospective study

Citing: Kim 2015 (10.01.18)

1. Chen 2009 – The impact of introducing medical emergency team system on the documentations of vital signs
   1. Citing: None (10.01.18)
2. Chen 2017 - Dynamic and personalized risk forecast in step-down units: Implications for monitoring paradigms
   1. Citing: Not found (10.01.18)
3. Churpek 2016 – The value of vital sign trends for detecting clinical deterioration on the wards
   1. Citing: None (10.01.18)
4. Considine 2013 - Emergency department rapid response systems: The case for a standardized approach to deteriorating patients
   1. Citing: None (10.01.18)
5. Derby - 2017 - Clinical Nurse Specialist-Driven Practice Change: Standardizing Vital Sign Monitoring
   1. Citing: None (10.01.18)
6. Devoe 2016 - Correlation of the predictive ability of early warning metrics and mortality for cardiac arrest patients receiving in-hospital Advanced Cardiovascular Life Support
   1. Citing: None (10.01.18)
7. Eggeman 2017
   1. Citing: Not found (11.01.18)
8. Escobar 2012 – Early detection of impending physiologic deterioration among patients who are not in intensive care: development of predictive models using data from an automated electronic medical record
   1. Citing: None (10.01.18)
9. Goldstein 2015 - Classifying Individuals Based on a Densely Captured Sequence of Vital Signs: An Example using Repeated Blood Pressure Measurements during Hemodialysis Treatment
   1. Citing: None (10.01.17)
10. Groarke 2008 – Use of an admission early warning score to predict patient morbidity and mortality and treatment success
    1. Citing: None (10.01.18)
11. Hudson 2015 – Early identification and management of the unstable adult patient in the emergency department
    1. Citing: None (10.01.18)
12. Kellett 2011 - Who will be sicker in the morning? Changes in the Simple Clinical Score the day after admission and the subsequent outcomes of acutely ill unselected medical patients
    1. Citing: Kellett 2013 (Medical) (10.01.2018)
13. Kellett 2014 – How to follow the NEWS
    1. Citing: Not found (10.01.2018)
14. Kellett 2015 – Trends in weighted vital signs and the clinical course of 44,531 acutely ill medical patients while in hospital
    1. Citing: Not found (10.01.2018)
15. Kellett 2013 - Changes and their prognostic implications in the abbreviated Vitalpac™ earlywarning score (ViEWS) after admission to hospital of 18,853 acutely ill medical patients
    1. Citing: Murray 2014 (10.01.2018)
16. Kellett 2013 – Changes and their prognostic implications in the abbreviated VitalPAC Early Warning Score (ViEWS) after admission to hospital of 18,827 surgical patients.
    1. Citing: None (10.01.2018)
17. Kim 2015 – Modified early warning score changes prior to cardiac arrest in general wards
    1. Citing: None (10.01.2018)
18. Ljunggren 2016 – The association between vital signs and mortality in a retrospective cohort study of an unselected emergency department population
    1. Citing: None (10.01.2018)
19. Mao 2011 – Medical Data Mining for Early Deterioration Warning in General Hospital Wards
    1. Citing: Not found (10.01.2018)
20. McGaughey 2007 – Outreach and Early Warning Systems (EWS) for the prevention of Intensive Care admission and death of critically ill adult patients on general hospital wards
    1. Citing: (09.01.2018)
21. Murray 2014 – Trajectories of the averaged abbreviated Vitalpac early warning score (AbEWS) and clinical course of 44,531 consecutive admissions hospitalized for acute medical illness.
    1. Citing: None (09.01.2018)
22. Nguyen 2016 – Predicting all-cause readmissions using electronic health record data from the entire hospitalization: Model development and comparison
    1. Citing: None (09.01.2018)
23. O'Connell 2016 – A newly designed observation and response chart's effect upon adverse inpatient outcomes and rapid response team activity
    1. Citing: None (09.01.2018)
24. Puskarich 2015 – Abstract: Persistence of tachycardia and tachypnea are associated with mortality in normotensive emergency department patients admitted to the hospital
    1. Citing: Not found (09.01.2018)
    2. Not included as reference, but tracking of this abstract yielded full article (Puskarich 2017)
25. Puskarich 2017 – Association between persistent tachycardia and tachypnea and in-hospital mortality among non-hypotensive emergency department patients admitted to the hospital.
    1. Citing: None (09.01.2018)
26. Quarterman 2005
    1. Citing: None (11.01.18)
27. Schmidt 2015 – Impact of introducing an electronic physiological surveillance system on hospital mortality
    1. Citing: None (09.01.2018)
28. Still 2017 - Predictors of Second Medical Emergency Team Activation Within 24 Hours of Index Event
    1. Citing: Not found (11.01.18)
29. Subbe 2013 – Not getting better means getting worse - Trends in Early Warning Scores suggest that there might only be a short time span to rescue those threatening to fall off a "physiological" cliff?
    1. Citing: None (09.01.2018)
30. Zimlichman 2012 – Early recognition of acutely deteriorating patients in non-intensive care units: Assessment of an innovative monitoring technology
    1. Citing: None (09.01.2018)
31. Wang 2017 – Early in-hospital clinical deterioration is not predicted by severity of illness, functional status, or comorbidity
    1. Citing: None (09.01.2018)
32. Wong 2011 – Does adding risk-trends to survival models improve in-hospital mortality predictions? A cohort study
    1. Citing: Churpek 2016 (09.01.2018)
