# Supplementary material for: The value of vital sign trends in predicting and monitoring clinical deterioration: A systematic review
Source: PLoS One. 2019 Jan 15;14(1):e0210875. doi: 10.1371/journal.pone.0210875 (PMC6333367; doi:10.1371/journal.pone.0210875)
Supplement: S4 Appendix — (DOCX) [file pone.0210875.s004.docx]

S4 Appendix: Full-text screening

Green: Fulfill inclusion, Red: Exclusion, Yellow: No information.

Abbreviations: EWS – early warning scores, ED – emergency department, MET – medical emergency team

|  |  |  |  |  |  | **Inclusion criteria** |  |  |  | **Exclusion criteria** |  |  |  |  |
| --- | --- | --- | --- | --- | --- | --- | --- | --- | --- | --- | --- | --- | --- | --- |
| **Title** | **Year**  **First author** | **Country** | **Identified through** | **Inclusion** | **Reason for exclusion** | **Acutely ill adult patients** | **Vital sign trends** | **Outreach to RRT, cardiac arrest, ICU-transfer or mortality** | **Readable language** | **Arrival ICU** | **< 100 patients** | **Study design** | **Only data on patients with specific condition or disease** | **No separate adult data** |
| Detecting dynamical changes in vital signs using switching Kalman filter [1] | 2017  Almeida | UK | Literature search |  | Not trend. Peer reviewed conference proceedings. |  |  |  |  |  |  | Cohort study |  |  |
| Variations in Vital Signs in the Last Days of Life in Patients With Advanced Cancer [2] | 2014  Bruera | USA | Recommended by expert |  | Patients with terminal cancer. |  |  |  |  |  |  | Cohort study |  |  |
| In-hospital mortality and morbidity of elderly medical patients can be predicted at admission by the Modified Early Warning Score: A prospective study [3] | 2009  Cei | Italy | Literature search |  | Not trend. |  |  |  |  |  |  | Cohort study |  |  |
| The impact of introducing medical emergency team system on the documentations of vital signs [4] | 2009  Chen J | Australia | Literature search |  | Not trend. |  |  |  |  |  |  | Cohort study |  |  |
| Dynamic and personalized risk forecast in step-down units: Implications for monitoring paradigms [5] | 2017  Chen L | USA | Literature search |  | Not trend.  Step-down unit patients. |  |  |  |  |  |  | Cohort study |  |  |
| Emergency department rapid response systems: The case for a standardized approach to deteriorating patients [6] | 2013 Considine | Australia  Narrative review | Literature search |  | Narrative review. |  |  |  |  |  |  | Narra-tive review. |  |  |
| The value of vital sign trends for detecting clinical deterioration on  the wards [7] | 2016  Churpek | USA | Literature search |  |  |  |  |  |  |  |  | Cohort study |  |  |
| Clinical Nurse Specialist-Driven Practice Change: Standardizing Vital Sign monitoring [8] | 2017  Derby | USA | Literature search |  | Not trend. |  |  |  |  |  |  | Cohort study |  |  |
| Correlation of the predictive ability of early warning metrics and mortality for cardiac arrest patients receiving in-hospital Advanced Cardiovascular Life Support [9] | 2016  DeVoe | USA | Literature search |  | Not trend.  Cardiac arrest patients. |  |  |  |  |  |  | Cohort study |  |  |
| Deaths within 48 hours of admission through the emergency department: Patient characteristics [10] | 2017 Eggeman | USA | Literature search |  | Abstract only. |  |  |  |  |  |  | Confer-ence abstract |  |  |
| Early detection of impending physiologic deterioration among patients who are not in intensive care: development of predictive models using data from an automated electronic medical record [11] | 2012  Escobar | USA | Literature search |  | Multiparameter risk stratification model.  Insufficient data. |  |  |  |  |  |  | Cohort study |  |  |
| Classifying Individuals Based on a Densely Captured Sequence of Vital Signs: An Example using Repeated Blood Pressure Measurements during Hemodialysis Treatment [12] | 2015  Goldstein | USA | Recommended by expert |  | Not trend. Hemodialysis patients. |  |  |  |  |  |  | Cohort study |  |  |
| Use of an admission early warning score to predict patient morbidity and mortality and treatment success [13] | 2008  Groarke | Ireland | Literature search |  | Trend in EWS. |  |  |  |  |  |  | Cohort study |  |  |
| Early identification and management of the unstable adult patient in the emergency department [14] | 2015  Hudson | Australia | Literature search |  | Not trend. |  |  |  |  |  |  | Cohort study |  |  |
| Who will be sicker in the morning? Changes in the Simple Clinical Score the day after admission and the subsequent outcomes of acutely ill unselected medical patients [15] | 2011  Kellett | Ireland | Citation tracking |  | Trend in Simple Clinical Score. |  |  |  |  |  |  | Cohort study |  |  |
| How to follow NEWS [16] | 2014  Kellett | Canada | Literature search |  | Trend in EWS. Same cohort as Kellett et al. [17] |  |  |  |  |  |  | Cohort study |  |  |
| Trends in weighted vital signs and the clinical course of 44,531 acutely ill medical patients while in hospital [17] | 2015  Kellett | Canada | Literature search |  |  |  |  |  |  |  |  | Cohort study |  |  |
| Changes and their prognostic implications in the abbreviated VitalPAC Early Warning Score (ViEWS) after admission to hospital of 18,827 surgical patients [18] | 2013  Kellett | Canada | Citation tracking |  | Trend in EWS. |  |  |  |  |  |  | Cohort study |  |  |
| Changes and their prognostic implications in the abbreviated Vitalpac™ early warning score (ViEWS) after admission to hospital of 18,853 acutely ill medical patients [19] | 2013  Kellett | Canada | Citation tracking |  | Trend in EWS. Same cohort as Kellett et al. [17] |  |  |  |  |  |  | Cohort study |  |  |
| Modified early warning score changes prior to cardiac arrest in general wards [20] | 2015 Kim | South Korea | Literature search |  | Trend in EWS in patients prior to cardiac arrest. |  |  |  |  |  |  | Cohort study |  |  |
| The association between vital signs and mortality in a retrospective cohort study of an unselected emergency department population [21] | 2016  Ljunggren | Sweden | Literature search |  | Not trend. |  |  |  |  |  |  | Cohort study |  |  |
| Medical Data Mining for Early Deterioration Warning in General Hospital Wards [22] | 2011  Mao | China | Citation tracking |  | Multiparameter risk stratification model. Insufficient data. |  |  |  |  |  |  | Cohort study |  |  |
| Outreach and Early Warning Systems (EWS) for the prevention of Intensive Care admission and death of critically ill adult patients on general hospital wards [23] | 2007  McGaughey | UK | Literature search |  | Not trend. |  |  |  |  |  |  | System-atic review |  |  |
| Trajectories of the averaged abbreviated Vitalpac early warning score (AbEWS) and clinical course of 44,531 consecutive admissions hospitalized for acute medical illness [24] | 2014  Murray | Ireland | Literature search |  | Trend in EWS. Same cohort as Kellett et al. [17] |  |  |  |  |  |  | Cohort study |  |  |
| Predicting all-cause readmissions using electronic health record data from the entire hospitalization: Model development and comparison [25] | 2016  Nguyen | USA | Literature search |  | Multiparameter model for 30-day readmission. Insufficient data. |  |  |  |  |  |  | Cohort study |  |  |
| A newly designed observation and response chart's effect upon adverse inpatient outcomes and rapid response team activity [26] | 2016  O’Connell | Australia | Literature search |  | Not trend. |  |  |  |  |  |  | Cohort study |  |  |
| Persistence of tachycardia and tachypnea are associated with mortality in normotensive emergency department patients admitted to the hospital [27] | 2015  Puskarich | USA | Literature search |  | Conference abstract of [28] |  |  |  |  |  |  | Confer-ence abstract |  |  |
| Association between persistent tachycardia and tachypnea and in-hospital mortality among non-hypotensive emergency department patients admitted to the hospital [28] | 2017  Puskarich | USA | Citation tracking |  | Normotensive ED patients. |  |  |  |  |  |  | Cohort study |  |  |
| Use of a patient information system to audit the introduction of modified early warning scoring [29] | 2005  Quarterman | UK | Citation tracking |  | Not trend. |  |  |  |  |  |  | Cohort study |  |  |
| Impact of introducing an electronic physiological surveillance system on hospital mortality [30] | 2015  Schmidt | UK | Literature search |  | Not trend. |  |  |  |  |  |  | Cohort study |  |  |
| Predictors of Second Medical Emergency Team Activation Within 24 Hours of Index Event [31] | 2017  Still | USA | Literature search |  | 24 h post MET activation |  |  |  |  |  |  | Cohort study |  |  |
| Not getting better means getting worse - Trends in Early Warning Scores suggest that there might only be a short time span to rescue those threatening to fall off a physiological cliff? [32] | 2013  Subbe | UK | Literature search |  | Editorial of [18] |  |  |  |  |  |  | Editorial |  |  |
| Early in-hospital clinical deterioration is not predicted by severity of illness, functional status, or comorbidity [33] | 2017  Wang | USA | Literature search |  | Trend in EWS. |  |  |  |  |  |  | Cohort study |  |  |
| Does adding risk-trends to survival models improve in-hospital mortality predictions? A cohort study [34] | 2011  Wong | Canada | Citation tracking |  | Not trend. |  |  |  |  |  |  | Cohort study |  |  |
| Early recognition of acutely deteriorating patients in non-intensive care units: Assessment of an innovative monitoring technology [35] | 2012  Zimlichman | USA | Literature search |  | Patients with acute respiratory condition |  |  |  |  |  |  | Cohort study |  |  |

**References**

1. Almeida V, Nabney IT. Detecting dynamical changes in vital signs using switching Kalman filter. Conf Proc IEEE Eng Med Biol Soc. 2017;2017:2223-6. Epub 2017/10/25. doi: 10.1109/embc.2017.8037296. PubMed PMID: 29060338.

2. Bruera S, Chisholm G, Santos RD, Crovador C, Bruera E, Hui D. Variations in Vital Signs in the Last Days of Life in Patients With Advanced Cancer. J Pain Symptom Manage. 2014;48(4):510-7. doi: <https://doi.org/10.1016/j.jpainsymman.2013.10.019>.

3. Cei M, Bartolomei C, Mumoli N. In-hospital mortality and morbidity of elderly medical patients can be predicted at admission by the Modified Early Warning Score: A prospective study. Int J Clin Pract. 2009;63(4):591-5. PubMed PMID: 354331029.

4. Chen J, Hillman K, Bellomo R, Flabouris A, Finfer S, Cretikos M. The impact of introducing medical emergency team system on the documentations of vital signs. Resuscitation. 2009;80(1):35-43. PubMed PMID: 50332885.

5. Chen L, Ogundele O, Clermont G, Hravnak M, Pinsky MR, Dubrawski AW. Dynamic and personalized risk forecast in step-down units: Implications for monitoring paradigms. Annals of the American Thoracic Society. 2017;14(3):384-91. PubMed PMID: 614753334.

6. Considine J, Jones D, Bellomo R. Emergency department rapid response systems: The case for a standardized approach to deteriorating patients. Eur J Emerg Med. 2013;20(6):375-81. PubMed PMID: 52398578.

7. Churpek MM, Adhikari R, Edelson DP. The value of vital sign trends for detecting clinical deterioration on the wards. Resuscitation. 2016;102:1-5. Epub 2016/02/24. doi: 10.1016/j.resuscitation.2016.02.005. PubMed PMID: 26898412; PubMed Central PMCID: PMCPMC4834231.

8. Derby KM, Hartung NA, Wolf SL, Zak HL, Evenson LK. Clinical Nurse Specialist-Driven Practice Change: Standardizing Vital Sign Monitoring. Clinical nurse specialist CNS. 2017;31(6):343-8. PubMed PMID: 619804763.

9. DeVoe B, Roth A, Maurer G, Tamuz M, Lesser M, Pekmezaris R, et al. Correlation of the predictive ability of early warning metrics and mortality for cardiac arrest patients receiving in-hospital Advanced Cardiovascular Life Support. Heart Lung. 2016;45(6):497-502. Epub 2016/10/05. doi: 10.1016/j.hrtlng.2016.08.010. PubMed PMID: 27697395.

10. Eggeman D, Chang K, Eilbert W. Deaths within 48 hours of admission through the emergency department: Patient characteristics. Acad Emerg Med. 2017;24:S269. PubMed PMID: 616279981.

11. Escobar GJ, LaGuardia JC, Turk BJ, Ragins A, Kipnis P, Draper D. Early detection of impending physiologic deterioration among patients who are not in intensive care: development of predictive models using data from an automated electronic medical record. J Hosp Med. 2012;7(5):388-95. Epub 2012/03/27. doi: 10.1002/jhm.1929. PubMed PMID: 22447632.

12. Goldstein BA, Chang TI, Winkelmayer WC. Classifying individuals based on a densely captured sequence of vital signs: An example using repeated blood pressure measurements during hemodialysis treatment. J Biomed Inform. 2015;57(Supplement C):219-24. doi: <https://doi.org/10.1016/j.jbi.2015.08.010>.

13. Groarke JD, Gallagher J, Stack J, Aftab A, Dwyer C, McGovern R, et al. Use of an admission early warning score to predict patient morbidity and mortality and treatment success. Emerg Med J. 2008;25(12):803-6. PubMed PMID: 352770921.

14. Hudson P, Ekholm J, Johnson M, Langdon R. Early identification and management of the unstable adult patient in the emergency department. J Clin Nurs. 2015;24(21-22):3138-46. PubMed PMID: 611786086.

15. Kellett J, Emmanuel A, Deane B. Who will be sicker in the morning? Changes in the Simple Clinical Score the day after admission and the subsequent outcomes of acutely ill unselected medical patients. Eur J Intern Med. 2011;22(4):375-81. doi: <https://doi.org/10.1016/j.ejim.2011.03.005>.

16. Kellett J, Murray A. How to follow the NEWS. Acute Med. 2014;13(3):104-7.

17. Kellett J, Murray A, Woodworth S, Huang W. Trends in weighted vital signs and the clinical course of 44,531 acutely ill medical patients while in hospital. Acute Med. 2015;14(1):3-9. Epub 2015/03/07. PubMed PMID: 25745643.

18. Kellett J, Wang F, Woodworth S, Huang W. Changes and their prognostic implications in the abbreviated VitalPAC Early Warning Score (ViEWS) after admission to hospital of 18,827 surgical patients. Resuscitation. 2013;84(4):471-6. Epub 2012/12/12. doi: 10.1016/j.resuscitation.2012.12.002. PubMed PMID: 23228559.

19. Kellett J, Woodworth S, Wang F, Huang W. Changes and their prognostic implications in the abbreviated Vitalpac™ early warning score (ViEWS) after admission to hospital of 18,853 acutely ill medical patients. Resuscitation. 2013;84(1):13-20. doi: <https://doi.org/10.1016/j.resuscitation.2012.08.331>.

20. Kim WY, Shin YJ, Lee JM, Huh JW, Koh Y, Lim CM, et al. Modified early warning score changes prior to cardiac arrest in general wards. PLoS One. 2015;10 (6) (no pagination)(e0130523). PubMed PMID: 605586094.

21. Ljunggren M, Castren M, Nordberg M, Kurland L. The association between vital signs and mortality in a retrospective cohort study of an unselected emergency department population. Scand J Trauma Resusc Emerg Med. 2016;24:21. PubMed PMID: 616126089.

22. Mao Y, Chen Y, Hackmann G, Chen M, Lu C, Kollef M, et al., editors. Medical Data Mining for Early Deterioration Warning in General Hospital Wards. 2011 IEEE 11th International Conference on Data Mining Workshops; 2011 11-11 Dec. 2011.

23. McGaughey J, Alderdice F, Fowler R, Kapila A, Mayhew A, Moutray M. Outreach and Early Warning Systems (EWS) for the prevention of Intensive Care admission and death of critically ill adult patients on general hospital wards. Cochrane Database Syst Rev. 2007;(3) (no pagination)(CD005529). PubMed PMID: 351805590.

24. Murray A, Kellett J, Huang W, Woodworth S, Wang F. Trajectories of the averaged abbreviated Vitalpac early warning score (AbEWS) and clinical course of 44,531 consecutive admissions hospitalized for acute medical illness. Resuscitation. 2014;85(4):544-8. Epub 2013/12/24. doi: 10.1016/j.resuscitation.2013.12.015. PubMed PMID: 24361459.

25. Nguyen OK, Makam AN, Clark C, Zhang S, Xie B, Velasco F, et al. Predicting all-cause readmissions using electronic health record data from the entire hospitalization: Model development and comparison. Journal of Hospital Medicine. 2016. PubMed PMID: 608793205.

26. O'Connell A, Flabouris A, Kim SW, Horwood C, Hakendorf P, Thompson CH. A newly designed observation and response chart's effect upon adverse inpatient outcomes and rapid response team activity. Intern Med J. 2016;46(8):909-16. PubMed PMID: 611871173.

27. Puskarich MA, Nandi U, Jones AE. Persistence of tachycardia and tachypnea are associated with mortality in normotensive emergency department patients admitted to the hospital. Acad Emerg Med. 2015;1):S217. PubMed PMID: 71879148.

28. Puskarich MA, Nandi U, Long BG, Jones AE. Association between persistent tachycardia and tachypnea and in-hospital mortality among non-hypotensive emergency department patients admitted to the hospital. Clinical and experimental emergency medicine. 2017;4(1):2-9. Epub 2017/04/25. doi: 10.15441/ceem.16.144. PubMed PMID: 28435896; PubMed Central PMCID: PMCPMC5385508.

29. Quarterman CP, Thomas AN, McKenna M, McNamee R. Use of a patient information system to audit the introduction of modified early warning scoring. J Eval Clin Pract. 2005;11(2):133-8. Epub 2005/04/09. doi: 10.1111/j.1365-2753.2005.00513.x. PubMed PMID: 15813711.

30. Schmidt PE, Meredith P, Prytherch DR, Watson D, Watson V, Killen RM, et al. Impact of introducing an electronic physiological surveillance system on hospital mortality. BMJ quality & safety. 2015;24(1):10-20. Epub 2014/09/25. doi: 10.1136/bmjqs-2014-003073. PubMed PMID: 25249636.

31. Still M, Vanderlaan J, Brown C, Gordon M, Graham K, Holder C, et al. Predictors of Second Medical Emergency Team Activation Within 24 Hours of Index Event. J Nurs Care Qual. 2017. Epub 2017/06/29. doi: 10.1097/ncq.0000000000000272. PubMed PMID: 28658191.

32. Subbe CP, Thorpe CM, Hancock C. Not getting better means getting worse - Trends in Early Warning Scores suggest that there might only be a short time span to rescue those threatening to fall off a "physiological" cliff? Resuscitation. 2013;84(4):409-10. PubMed PMID: 52460035.

33. Wang J, Hahn SS, Kline M, Cohen RI. Early in-hospital clinical deterioration is not predicted by severity of illness, functional status, or comorbidity. Int J Gen Med. 2017;10:329-34. PubMed PMID: 619212411.

34. Wong J, Taljaard M, Forster AJ, van Walraven C. Does adding risk-trends to survival models improve in-hospital mortality predictions? A cohort study. BMC Health Serv Res. 2011;11(1):171. doi: 10.1186/1472-6963-11-171.

35. Zimlichman E, Szyper-Kravitz M, Shinar Z, Klap T, Levkovich S, Unterman A, et al. Early recognition of acutely deteriorating patients in non-intensive care units: Assessment of an innovative monitoring technology. J Hosp Med. 2012;7(8):628-33. PubMed PMID: 52150095.
