# Supplementary material for: The value of vital sign trends in predicting and monitoring clinical deterioration: A systematic review
Source: PLoS One. 2019 Jan 15;14(1):e0210875. doi: 10.1371/journal.pone.0210875 (PMC6333367; doi:10.1371/journal.pone.0210875)
Supplement: S5 Appendix — (DOCX) [file pone.0210875.s005.docx]

| ***Study: Churpek et al. 2016 [1]*** | |
| --- | --- |
| **Study Participation** |  |
| *Key characteristics of source population* | Yes |
| *Method used to identify population* | Yes |
| *Recruitment period* | Yes |
| *Place of recruitment* | Yes |
| *Adequate study participation by eligible individuals* | Yes |
| *Baseline study sample characteristics* | Partly |
| Summarized risk of potential bias | Low |
| **Study Attrition** |  |
| *Response rate* | Yes |
| *Attempts to collect information on participants who dropped out described* | No |
| *Reasons for loss to follow-up are provided.* | No |
| *Participants lost to follow-up are adequately described for key characteristics.* | No |
| *There are no important differences between key characteristics and outcomes in participants who completed the study and those who did not.* | Unsure |
| Summarized risk of potential bias | Moderate |
| **Prognostic Factor Measurement** |  |
| *A clear definition or description of the prognostic factor measured is provided.* | Yes |
| *Continuous variables are reported or appropriate (i.e., not data-dependent) cut-points are used.* | Yes |
| *The prognostic factor measure and method are adequately valid and reliable to limit misclassification bias.* | Yes |
| *Adequate proportion of the study sample has complete data for prognostic factors.* | Unsure |
| *The method and setting of measurement are the same for all study participants.* | Yes |
| *Appropriate methods are used if imputation is used for missing prognostic factor data.* | Yes |
| Summarized risk of potential bias | Low |
| **Outcome Measurement** |  |
| *Definition of the outcome* | Yes |
| *Valid and reliable measurement of outcome to limit misclassification bias* | Yes |
| *The method and setting of measurement are the same for all study participants.* | Yes |
| Summarized risk of potential bias | Low |
|  |  |
| **Study Confounding** |  |
| *All important confounders measured* | Partly |
| *Clear definitions of the important confounders measured are provided* | No |
| *Valid and reliable measurement of confounders* | Unsure |
| *The method and setting of confounding measurement are the same for all study participants.* | Yes |
| *Appropriate methods are used if imputation is used for missing confounder data.* | Unsure |
| *Appropriate accounting for confounding in the study design and the analysis* | No |
| Summarized risk of potential bias | Moderate |
| **Statistical Analysis** |  |
| *There is sufficient presentation of data to assess the adequacy of the analysis.* | Partly |
| *Model development strategy adequate for the study design.* | Yes |
| *There is no selective reporting of results.* | Yes |
| Summarized risk of potential bias | Moderate |
| **Overall** | **Moderate** |
| **Table for QUIPS-assessment based on ”Table 3: Guidelines for Assessing Quality in Prognostic Studies on the Basis of Framework of Potential Biases” by Hayden et al. [2]** | |

| **GRADE criteria** | **Rating** | **Footnotes** | **Quality of evidence** |
| --- | --- | --- | --- |
| **Study design** | No  Serious (-1)  Very serious (-2) | Observational study: Low |  |
| **Risk of Bias** | No  Serious (-1)  Very serious (-2) | Moderate risk of bias, assessed using QUIPS. |  |
| **Inconsistency** | No  Serious (-1)  Very serious (-2) | Not applicable - only one study per outcome. |  |
| **Indirectness** | No  Serious (-1)  Very serious (-2) | No | ⊕⊕••  Low |
| **Imprecision** | No  Serious (-1)  Very serious (-2) | No. Narrow confidence intervals - although not all CI 95% given. |  |
| **Publication Bias** | Undetected  Strongly suspected (-1) | Undetected |  |
| **Other** | Large effect (+1 or +2)  Dose Response (+1 or +2)  No Plausible confounding  (+1 or +2) | Not applicable |  |

GRADE-assessment of Churpek et al.

| ***Study: Kellett et al. 2015 [3]*** | |
| --- | --- |
| **Study Participation** |  |
| *Key characteristics of source population* | Yes |
| *Method used to identify population* | Yes |
| *Recruitment period* | Yes |
| *Place of recruitment* | Yes |
| *Adequate study participation by eligible individuals* | Yes |
| *Baseline study sample characteristics* | Partly |
| Summarized risk of potential bias | Low |
| **Study Attrition** |  |
| *Response rate* | Yes |
| *Attempts to collect information on participants who dropped out described* | No |
| *Reasons for loss to follow-up are provided.* | No |
| *Participants lost to follow-up are adequately described for key characteristics.* | No |
| *There are no important differences between key characteristics and outcomes in participants who completed the study and those who did not.* | Unsure |
| Summarized risk of potential bias | Moderate |
| **Prognostic Factor Measurement** |  |
| *A clear definition or description of the prognostic factor measured is provided.* | Yes |
| *Continuous variables are reported or appropriate (i.e., not data-dependent) cut-points are used.* | Yes |
| *The prognostic factor measure and method are adequately valid and reliable to limit misclassification bias.* | Yes |
| *Adequate proportion of the study sample has complete data for prognostic factors.* | Yes |
| *The method and setting of measurement are the same for all study participants.* | Yes |
| *Appropriate methods are used if imputation is used for missing prognostic factor data.* | Yes |
| Summarized risk of potential bias | Low |
| **Outcome Measurement** |  |
| *Definition of the outcome* | Yes |
| *Valid and reliable measurement of outcome to limit misclassification bias* | Yes |
| *The method and setting of measurement are the same for all study participants.* | Yes |
| Summarized risk of potential bias | Low |
|  |  |
| **Study Confounding** |  |
| *All important confounders measured* | Partly |
| *Clear definitions of the important confounders measured are provided* | No |
| *Valid and reliable measurement of confounders* | No |
| *The method and setting of confounding measurement are the same for all study participants.* | Yes |
| *Appropriate methods are used if imputation is used for missing confounder data.* | Yes |
| *Appropriate accounting for confounding in the study design and the analysis* | No |
| Summarized risk of potential bias | Moderate |
| **Statistical Analysis** |  |
| *There is sufficient presentation of data to assess the adequacy of the analysis.* | Yes |
| *Model development strategy adequate for the study design.* | Yes |
| *There is no selective reporting of results.* | Yes |
| Summarized risk of potential bias | Low |
| **Overall** | **Moderate** |

| **GRADE criteria** | **Rating** | **Footnotes** | **Quality of evidence** |
| --- | --- | --- | --- |
| **Study design** | No  Serious (-1)  Very serious (-2) | Observational study: Low |  |
| **Risk of Bias** | No  Serious (-1)  Very serious (-2) | Moderate risk of bias, assessed using QUIPS. |  |
| **Inconsistency** | No  Serious (-1)  Very serious (-2) | Not applicable - only one study per outcome |  |
| **Indirectness** | No  Serious (-1)  Very serious (-2) | No | ⊕•••  Very Low |
| **Imprecision** | No  Serious (-1)  Very serious (-2) | Serious. Large standard deviations. No statistical significance. | Downgraded due to serious risk of imprecision. |
| **Publication Bias** | Undetected  Strongly suspected (-1) | Undetected |  |
| **Other** | Large effect (+1 or +2)  Dose Response (+1 or +2)  No Plausible confounding  (+1 or +2) | Not applicable |  |

GRADE-assessment of Kellett et al. 2015

| **Study: Groarke et al. 2008 [4]** | |
| --- | --- |
| **Study Participation** |  |
| *Key characteristics of source population* | Yes |
| *Method used to identify population* | Yes |
| *Recruitment period* | Yes |
| *Place of recruitment* | Yes |
| *Adequate study participation by eligible individuals* | Partly |
| *Baseline study sample characteristics* | Yes |
| Summarized risk of potential bias | Moderate |
| **Study Attrition** |  |
| *Response rate* | Yes |
| *Attempts to collect information on participants who dropped out described* | Not applicable |
| *Reasons for loss to follow-up are provided.* | Not applicable |
| *Participants lost to follow-up are adequately described for key characteristics.* | Not applicable |
| *There are no important differences between key characteristics and outcomes in participants who completed the study and those who did not.* | Not applicable |
| Summarized risk of potential bias | Low |
| **Prognostic Factor Measurement** |  |
| *A clear definition or description of the prognostic factor measured is provided.* | Yes |
| *Continuous variables are reported or appropriate (i.e., not data-dependent) cut-points are used.* | Yes |
| *The prognostic factor measure and method are adequately valid and reliable to limit misclassification bias.* | Yes |
| *Adequate proportion of the study sample has complete data for prognostic factors.* | Yes |
| *The method and setting of measurement are the same for all study participants.* | Yes |
| *Appropriate methods are used if imputation is used for missing prognostic factor data.* | Partly |
| Summarized risk of potential bias | Moderate |
| **Outcome Measurement** |  |
| *Definition of the outcome* | Yes |
| *Valid and reliable measurement of outcome to limit misclassification bias* | Yes |
| *The method and setting of measurement are the same for all study participants.* | Yes |
| Summarized risk of potential bias | Low |
|  |  |
| **Study Confounding** |  |
| *All important confounders measured* | Partly |
| *Clear definitions of the important confounders measured are provided* | Partly |
| *Valid and reliable measurement of confounders* | Partly |
| *The method and setting of confounding measurement are the same for all study participants.* | Yes |
| *Appropriate methods are used if imputation is used for missing confounder data.* | Unsure |
| *Appropriate accounting for confounding in the study design and the analysis* | Partly |
| Summarized risk of potential bias | Moderate |
| **Statistical Analysis** |  |
| *There is sufficient presentation of data to assess the adequacy of the analysis.* | Yes |
| *Model development strategy adequate for the study design.* | Yes |
| *There is no selective reporting of results.* | Yes |
| Summarized risk of potential bias | Low |
| **Overall** | **Moderate** |

| **GRADE criteria** | **Rating** | **Footnotes** | **Quality of evidence** |
| --- | --- | --- | --- |
| **Study design** | No  Serious (-1)  Very serious (-2) | Observational study: Low |  |
| **Risk of Bias** | No  Serious (-1)  Very serious (-2) | Moderate risk of bias, assessed using QUIPS. |  |
| **Inconsistency** | No  Serious (-1)  Very serious (-2) | Not applicable (only one study per outcome) |  |
| **Indirectness** | No  Serious (-1)  Very serious (-2) | No | ⊕•••  Very Low |
| **Imprecision** | No  Serious (-1)  Very serious (-2) | Serious. Few patients. Particularly in higher score categories. Low number of events. | Downgraded due to serious risk of imprecision. |
| **Publication Bias** | Undetected  Strongly suspected (-1) | Undetected |  |
| **Other** | Large effect (+1 or +2)  Dose Response (+1 or +2)  No Plausible confounding  (+1 or +2) | Not applicable |  |

GRADE-assessment of Groarke et al.

| **Study: Kellett et al. 2011 [5]** | |
| --- | --- |
| **Study Participation** |  |
| *The source population or population of interest is adequately described for key characteristics.* | Yes |
| *Method used to identify population* | Yes |
| *Recruitment period* | Yes |
| *Place of recruitment* | Yes |
| *Adequate study participation by eligible individuals* | Partly |
| *Baseline study sample characteristics* | Partly |
| Summarized risk of potential bias | Moderate |
| **Study Attrition** |  |
| *Response rate* | Partly |
| *Attempts to collect information on participants who dropped out described* | Yes |
| *Reasons for loss to follow-up are provided.* | Yes |
| *Participants lost to follow-up are adequately described for key characteristics.* | Partly |
| *There are no important differences between key characteristics and outcomes in participants who completed the study and those who did not.* | Unsure |
| Summarized risk of potential bias | Moderate |
| **Prognostic Factor Measurement** |  |
| *A clear definition or description of the prognostic factor measured is provided.* | Yes |
| *Continuous variables are reported or appropriate (i.e., not data-dependent) cut-points are used.* | Yes |
| *The prognostic factor measure and method are adequately valid and reliable to limit misclassification bias.* | Yes |
| *Adequate proportion of the study sample has complete data for prognostic factors.* | Yes |
| *The method and setting of measurement are the same for all study participants.* | Yes |
| *Appropriate methods are used if imputation is used for missing prognostic factor data.* | Yes |
| Summarized risk of potential bias | Low |
| **Outcome Measurement** |  |
| *Definition of the outcome* | Yes |
| *Valid and reliable measurement of outcome to limit misclassification bias* | Yes |
| *The method and setting of measurement are the same for all study participants.* | Yes |
| Summarized risk of potential bias | Low |
|  |  |
| **Study Confounding** |  |
| *All important confounders measured* | Partly |
| *Clear definitions of the important confounders measured are provided* | Yes |
| *Valid and reliable measurement of confounders* | Partly |
| *The method and setting of confounding measurement are the same for all study participants.* | Yes |
| *Appropriate methods are used if imputation is used for missing confounder data.* | Unsure |
| *Appropriate accounting for confounding in the study design and the analysis* | Partly |
| Summarized risk of potential bias | Moderate |
| **Statistical Analysis** |  |
| *There is sufficient presentation of data to assess the adequacy of the analysis.* | Yes |
| *Model development strategy adequate for the study design.* | Yes |
| *There is no selective reporting of results.* | Yes |
| Summarized risk of potential bias | Low |
| **Overall** | **Moderate** |

| **GRADE criteria** | **Rating** | **Footnotes** | **Quality of evidence** |
| --- | --- | --- | --- |
| **Study design** | No  Serious (-1)  Very serious (-2) | Observational study: Low |  |
| **Risk of Bias** | No  Serious (-1)  Very serious (-2) | Moderate risk of bias, assessed using QUIPS. |  |
| **Inconsistency** | No  Serious (-1)  Very serious (-2) | Not applicable - only one study per outcome. |  |
| **Indirectness** | No  Serious (-1)  Very serious (-2) | No | ⊕•••  Very Low |
| **Imprecision** | No  Serious (-1)  Very serious (-2) | Serious. Low number of events. | Downgraded due to serious risk of imprecision. |
| **Publication Bias** | Undetected  Strongly suspected (-1) | Undetected |  |
| **Other** | Large effect (+1 or +2)  Dose Response (+1 or +2)  No Plausible confounding  (+1 or +2) | Not applicable |  |

GRADE-assessment of Kellett et al. 2011

| **Study: Kellett et al. 2013 [6]** | |
| --- | --- |
| **Study Participation** |  |
| *Key characteristics of source population* | Yes |
| *Method used to identify population* | Yes |
| *Recruitment period* | Yes |
| *Place of recruitment* | Yes |
| *Adequate study participation by eligible individuals* | Yes |
| *Baseline study sample characteristics* | Partly |
| Summarized risk of potential bias | Low |
| **Study Attrition** |  |
| *Response rate* | Yes |
| *Attempts to collect information on participants who dropped out described* | No |
| *Reasons for loss to follow-up are provided.* | Yes |
| *Participants lost to follow-up are adequately described for key characteristics.* | No |
| *There are no important differences between key characteristics and outcomes in participants who completed the study and those who did not.* | Unsure |
| Summarized risk of potential bias | Moderate |
| **Prognostic Factor Measurement** |  |
| *A clear definition or description of the prognostic factor measured is provided.* | Yes |
| *Continuous variables are reported or appropriate (i.e., not data-dependent) cut-points are used.* | Yes |
| *The prognostic factor measure and method are adequately valid and reliable to limit misclassification bias.* | Yes |
| *Adequate proportion of the study sample has complete data for prognostic factors.* | Yes |
| *The method and setting of measurement are the same for all study participants.* | Yes |
| *Appropriate methods are used if imputation is used for missing prognostic factor data.* | Yes |
| Summarized risk of potential bias | Low |
| **Outcome Measurement** |  |
| *Definition of the outcome* | Yes |
| *Valid and reliable measurement of outcome to limit misclassification bias* | Yes |
| *The method and setting of measurement are the same for all study participants.* | Yes |
| Summarized risk of potential bias | Low |
|  |  |
| **Study Confounding** |  |
| *All important confounders measured* | Partly |
| *Clear definitions of the important confounders measured are provided* | No |
| *Valid and reliable measurement of confounders* | No |
| *The method and setting of confounding measurement are the same for all study participants.* | Yes |
| *Appropriate methods are used if imputation is used for missing confounder data.* | Unsure |
| *Appropriate accounting for confounding in the study design and the analysis* | No |
| Summarized risk of potential bias | Moderate |
| **Statistical Analysis** |  |
| *There is sufficient presentation of data to assess the adequacy of the analysis.* | Yes |
| *Model development strategy adequate for the study design.* | Yes |
| *There is no selective reporting of results.* | Yes |
| Summarized risk of potential bias | Low |
| **Overall** | **Moderate** |

| **GRADE criteria** | **Rating** | **Footnotes** | **Quality of evidence** |
| --- | --- | --- | --- |
| **Study design** | No  Serious (-1)  Very serious (-2) | Observational study: Low |  |
| **Risk of Bias** | No  Serious (-1)  Very serious (-2) | Low risk of bias, assessed using QUIPS. |  |
| **Inconsistency** | No  Serious (-1)  Very serious (-2) | Not applicable - only one study per outcome. |  |
| **Indirectness** | No  Serious (-1)  Very serious (-2) | No | ⊕•••  Very Low |
| **Imprecision** | No  Serious (-1)  Very serious (-2) | Serious. Low number of events. | Downgraded due to serious risk of imprecision. |
| **Publication Bias** | Undetected  Strongly suspected (-1) | Undetected |  |
| **Other** | Large effect (+1 or +2)  Dose Response (+1 or +2)  No Plausible confounding  (+1 or +2) | Not applicable |  |

GRADE-assessment of Kellett et al. 2013.

| **Study: Wang et al. 2017 [7]** | |
| --- | --- |
| **Study Participation** |  |
| *The source population or population of interest is adequately described for key characteristics.* | Yes |
| *Method used to identify population* | Yes |
| *Recruitment period* | Yes |
| *Place of recruitment* | Yes |
| *Adequate study participation by eligible individuals* | Yes |
| *Baseline study sample characteristics* | Yes |
| Summarized risk of potential bias | Low |
| **Study Attrition** |  |
| *Response rate* | Yes |
| *Attempts to collect information on participants who dropped out described* | Not applicable |
| *Reasons for loss to follow-up are provided.* | Not applicable |
| *Participants lost to follow-up are adequately described for key characteristics.* | Not applicable |
| *There are no important differences between key characteristics and outcomes in participants who completed the study and those who did not.* | Not applicable |
| Summarized risk of potential bias | Low |
| **Prognostic Factor Measurement** |  |
| *A clear definition or description of the prognostic factor measured is provided.* | Yes |
| *Continuous variables are reported or appropriate (i.e., not data-dependent) cut-points are used.* | Yes |
| *The prognostic factor measure and method are adequately valid and reliable to limit misclassification bias.* | Yes |
| *Adequate proportion of the study sample has complete data for prognostic factors.* | Yes |
| *The method and setting of measurement are the same for all study participants.* | Yes |
| *Appropriate methods are used if imputation is used for missing prognostic factor data.* | Unsure |
| Summarized risk of potential bias | Low |
| **Outcome Measurement** |  |
| *Definition of the outcome* | Yes |
| *Valid and reliable measurement of outcome to limit misclassification bias* | Partly |
| *The method and setting of measurement are the same for all study participants.* | Yes |
| Summarized risk of potential bias | Low |
|  |  |
| **Study Confounding** |  |
| *All important confounders measured* | Partly |
| *Clear definitions of the important confounders measured are provided* | Partly |
| *Valid and reliable measurement of confounders* | Unsure |
| *The method and setting of confounding measurement are the same for all study participants.* | Yes |
| *Appropriate methods are used if imputation is used for missing confounder data.* | Unsure |
| *Appropriate accounting for confounding in the study design and the analysis* | Partly |
| Summarized risk of potential bias | Moderate |
| **Statistical Analysis** |  |
| *There is sufficient presentation of data to assess the adequacy of the analysis.* | Yes |
| *Model development strategy adequate for the study design.* | Yes |
| *There is no selective reporting of results.* | Yes |
| Summarized risk of potential bias | Low |
| **Overall** | **Moderate** |

| **GRADE criteria** | **Rating** | **Footnotes** | **Quality of evidence** |
| --- | --- | --- | --- |
| **Study design** | No  Serious (-1)  Very serious (-2) | Observational study: Low |  |
| **Risk of Bias** | No  Serious (-1)  Very serious (-2) | Moderate risk of bias, assessed using QUIPS. |  |
| **Inconsistency** | No  Serious (-1)  Very serious (-2) | Not applicable - only one study per outcome. |  |
| **Indirectness** | No  Serious (-1)  Very serious (-2) | No | ⊕•••  Very Low |
| **Imprecision** | No  Serious (-1)  Very serious (-2) | Serious. Low number of events. | Downgraded due to serious risk of imprecision. |
| **Publication Bias** | Undetected  Strongly suspected (-1) | Undetected |  |
| **Other** | Large effect (+1 or +2)  Dose Response (+1 or +2)  No Plausible confounding  (+1 or +2) | Not applicable |  |

GRADE-assessment of Wang et al.

**References**

1. Churpek MM, Adhikari R, Edelson DP. The value of vital sign trends for detecting clinical deterioration on the wards. Resuscitation. 2016;102:1-5. Epub 2016/02/24. doi: 10.1016/j.resuscitation.2016.02.005. PubMed PMID: 26898412; PubMed Central PMCID: PMCPMC4834231.

2. Hayden JA, Côté P, Bombardier C. Evaluation of the quality of prognosis studies in systematic reviews. Ann Intern Med. 2006;144(6):427-37. doi: 10.7326/0003-4819-144-6-200603210-00010.

3. Kellett J, Murray A, Woodworth S, Huang W. Trends in weighted vital signs and the clinical course of 44,531 acutely ill medical patients while in hospital. Acute Med. 2015;14(1):3-9. Epub 2015/03/07. PubMed PMID: 25745643.

4. Groarke JD, Gallagher J, Stack J, Aftab A, Dwyer C, McGovern R, et al. Use of an admission early warning score to predict patient morbidity and mortality and treatment success. Emerg Med J. 2008;25(12):803-6. PubMed PMID: 352770921.

5. Kellett J, Emmanuel A, Deane B. Who will be sicker in the morning? Changes in the Simple Clinical Score the day after admission and the subsequent outcomes of acutely ill unselected medical patients. Eur J Intern Med. 2011;22(4):375-81. doi: <https://doi.org/10.1016/j.ejim.2011.03.005>.

6. Kellett J, Wang F, Woodworth S, Huang W. Changes and their prognostic implications in the abbreviated VitalPAC Early Warning Score (ViEWS) after admission to hospital of 18,827 surgical patients. Resuscitation. 2013;84(4):471-6. Epub 2012/12/12. doi: 10.1016/j.resuscitation.2012.12.002. PubMed PMID: 23228559.

7. Wang J, Hahn SS, Kline M, Cohen RI. Early in-hospital clinical deterioration is not predicted by severity of illness, functional status, or comorbidity. Int J Gen Med. 2017;10:329-34. PubMed PMID: 619212411.
